# Tankyrase-1 regulates RBP-mediated mRNA turnover to promote muscle fiber formation

**DOI:** 10.1093/nar/gkae059

**Published:** 2024-02-07

**Authors:** Souad Mubaid, Brenda Janice Sanchez, Rinad A Algehani, Viktoriia Skopenkova, Pauline Adjibade, Derek T Hall, Sandrine Busque, Xian Jin Lian, Kholoud Ashour, Anne-Marie K Tremblay, Graeme Carlile, Jean-Philippe Gagné, Andrea Diaz-Gaxiola, Shahryar Khattak, Sergio Di Marco, David Y Thomas, Guy G Poirier, Imed-Eddine Gallouzi

**Affiliations:** Dept. of Biochemistry, McGill University, 3655 Promenade Sir William Osler, Montreal, QC H3G 1Y6, Canada; Rosalind & Morris Goodman Cancer Institute, McGill University, 1160 Pine Avenue, Montreal, QC H3A 1A3, Canada; KAUST Smart-Health Initiative (KSHI) and Biological and Environmental Science and Engineering (BESE) Division, King Abdullah University of Science and Technology (KAUST), Jeddah, Saudi Arabia; KAUST Smart-Health Initiative (KSHI) and Biological and Environmental Science and Engineering (BESE) Division, King Abdullah University of Science and Technology (KAUST), Jeddah, Saudi Arabia; KAUST Smart-Health Initiative (KSHI) and Biological and Environmental Science and Engineering (BESE) Division, King Abdullah University of Science and Technology (KAUST), Jeddah, Saudi Arabia; Dept. of Biochemistry, McGill University, 3655 Promenade Sir William Osler, Montreal, QC H3G 1Y6, Canada; Rosalind & Morris Goodman Cancer Institute, McGill University, 1160 Pine Avenue, Montreal, QC H3A 1A3, Canada; Dept. of Biochemistry, McGill University, 3655 Promenade Sir William Osler, Montreal, QC H3G 1Y6, Canada; Rosalind & Morris Goodman Cancer Institute, McGill University, 1160 Pine Avenue, Montreal, QC H3A 1A3, Canada; Dept. of Biochemistry, McGill University, 3655 Promenade Sir William Osler, Montreal, QC H3G 1Y6, Canada; Rosalind & Morris Goodman Cancer Institute, McGill University, 1160 Pine Avenue, Montreal, QC H3A 1A3, Canada; Dept. of Biochemistry, McGill University, 3655 Promenade Sir William Osler, Montreal, QC H3G 1Y6, Canada; Rosalind & Morris Goodman Cancer Institute, McGill University, 1160 Pine Avenue, Montreal, QC H3A 1A3, Canada; Dept. of Biochemistry, McGill University, 3655 Promenade Sir William Osler, Montreal, QC H3G 1Y6, Canada; Rosalind & Morris Goodman Cancer Institute, McGill University, 1160 Pine Avenue, Montreal, QC H3A 1A3, Canada; Dept. of Biochemistry, McGill University, 3655 Promenade Sir William Osler, Montreal, QC H3G 1Y6, Canada; Rosalind & Morris Goodman Cancer Institute, McGill University, 1160 Pine Avenue, Montreal, QC H3A 1A3, Canada; Dept. of Biochemistry, McGill University, 3655 Promenade Sir William Osler, Montreal, QC H3G 1Y6, Canada; Centre de recherche du CHU de Québec-Pavillon CHUL, Faculté de Médecine, Université Laval, Québec G1V 4G2, Canada; KAUST Smart-Health Initiative (KSHI) and Biological and Environmental Science and Engineering (BESE) Division, King Abdullah University of Science and Technology (KAUST), Jeddah, Saudi Arabia; KAUST Smart-Health Initiative (KSHI) and Biological and Environmental Science and Engineering (BESE) Division, King Abdullah University of Science and Technology (KAUST), Jeddah, Saudi Arabia; KAUST Smart-Health Initiative (KSHI) and Biological and Environmental Science and Engineering (BESE) Division, King Abdullah University of Science and Technology (KAUST), Jeddah, Saudi Arabia; Dept. of Biochemistry, McGill University, 3655 Promenade Sir William Osler, Montreal, QC H3G 1Y6, Canada; Rosalind & Morris Goodman Cancer Institute, McGill University, 1160 Pine Avenue, Montreal, QC H3A 1A3, Canada; Dept. of Biochemistry, McGill University, 3655 Promenade Sir William Osler, Montreal, QC H3G 1Y6, Canada; Centre de recherche du CHU de Québec-Pavillon CHUL, Faculté de Médecine, Université Laval, Québec G1V 4G2, Canada; KAUST Smart-Health Initiative (KSHI) and Biological and Environmental Science and Engineering (BESE) Division, King Abdullah University of Science and Technology (KAUST), Jeddah, Saudi Arabia; Dept. of Biochemistry, McGill University, 3655 Promenade Sir William Osler, Montreal, QC H3G 1Y6, Canada; Rosalind & Morris Goodman Cancer Institute, McGill University, 1160 Pine Avenue, Montreal, QC H3A 1A3, Canada

## Abstract

Poly(ADP-ribosylation) (PARylation) is a post-translational modification mediated by a subset of ADP-ribosyl transferases (ARTs). Although PARylation-inhibition based therapies are considered as an avenue to combat debilitating diseases such as cancer and myopathies, the role of this modification in physiological processes such as cell differentiation remains unclear. Here, we show that Tankyrase1 (TNKS1), a PARylating ART, plays a major role in myogenesis, a vital process known to drive muscle fiber formation and regeneration. Although all *bona fide* PARPs are expressed in muscle cells, experiments using siRNA-mediated knockdown or pharmacological inhibition show that TNKS1 is the enzyme responsible of catalyzing PARylation during myogenesis. Via this activity, TNKS1 controls the turnover of mRNAs encoding myogenic regulatory factors such as *nucleophosmin* (NPM) and *myogenin*. TNKS1 mediates these effects by targeting RNA-binding proteins such as Human Antigen R (HuR). HuR harbors a conserved TNKS-binding motif (TBM), the mutation of which not only prevents the association of HuR with TNKS1 and its PARylation, but also precludes HuR from regulating the turnover of *NPM* and *myogenin* mRNAs as well as from promoting myogenesis. Therefore, our data uncover a new role for TNKS1 as a key modulator of RBP-mediated post-transcriptional events required for vital processes such as myogenesis.

## Graphical Abstract

**Graphical Abstract:**
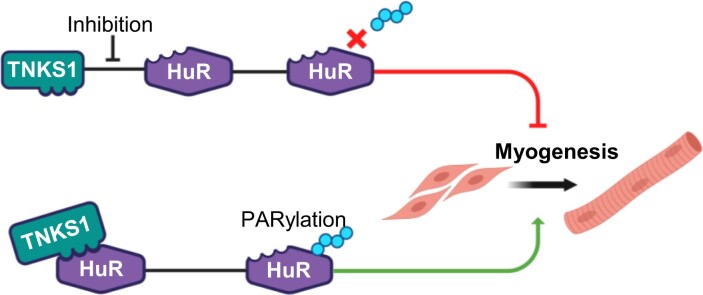

## Introduction

The covalent addition of functional groups is one of the well-characterized post-translational modifications (PTMs) used by the cell to modulate and expand the function of its protein network. There are >400 different types of PTMs affecting many aspects of protein functions. During the last few decades, the list of PTMs has expanded to include phosphorylation, glycosylation, ubiquitination, SUMOylation, nitrosylation, methylation, acetylation, lipidation, as well as poly(ADP-ribosylation) (PARylation) (1–10). The majority of these PTMs have been associated with numerous physiological and pathological phenotypes. Yet, our understanding of the role and impact of PARylation in cell homeostasis and physiology is quite limited (9,10). The importance of PARylation is underscored, however, by the fact that it is ubiquitous in nature as well as by numerous *in vitro* and *in vivo* studies highlighting the benefit of PARylation inhibition for the treatment of diseases such as cancer, muscle myopathies and some metabolic disorders (9–11). Recent clinical trials using these inhibitors nonetheless showed limited successes in combatting diseases such as diabetes as well as prostate, and colon cancers (9–11). Therefore, to design wider and efficient PARylation-inhibition-based therapies, a better understanding of the role of PARylation in vital processes such as cell homeostasis, metabolism, and differentiation is needed.

PARylation is a reversible PTM that is mediated by the hydrolysis of NAD^+^ and the transfer of ADP-ribose moieties to protein acceptors (12,13). This catalytic reaction is mediated by a subset of enzymes called poly(ADP-ribose) polymerases (PARPs) which are part of the ADP-ribosyl transferases (ARTDs) family of proteins (14,15). Although the majority of these enzymes transfer a single ADP-ribose (ADPr) moiety (i.e. mono(ADP-ribosyl) transferases or MARTs) or are catalytically inactive (16,17) four members, PARP1 (ARTD1), PARP2 (ARTD2) as well as PARP5a (ARTD5) and PARP5b (ARTD6) (also called tankyrase-1 (TNKS1) and -2 (TNKS2) respectively) exhibit PARylation activity by adding a polymer composed of up to 200 ADP-ribose subunits (termed poly(ADP-ribose); pADPr) to target proteins. These four PARPs are designated as “*bona fide* PARPs’’ since, as their name suggests, they synthesize polymers of ADPr (16,18–22). pADPr was initially characterized as a nucleic-acid-like molecule that is a product of a nuclear enzymatic activity (12). However, it was later discovered that PARylation is a post-translational modification capable of reprogramming the biochemical properties and activities of target proteins. It has been shown to regulate the interaction of target proteins with nucleic acids (23–25) and protein ligands (23,26–29) [by affecting their ubiquitination (29–32) or their cellular localization (27,33)].

The function of PARPs is essential since the double knockout of PARP1/PARP2 or TNKS1/TNKS2 in mice is embryonically lethal (34,35). They are known to play a role in several cellular processes including genomic maintenance, DNA damage response, transcription, and inflammation (23,25,36–39). While PARP1 is generally associated with actively transcribed genes (40,41), binding to nucleosomes and the DNA-damage response, PARP2 was shown to be involved, among other things, in lipid metabolism and in autophagosomes clearance (26,42–44). TNKS1 and TNKS2, on the other hand, have a well-established role in regulating telomere maintenance, canonical Wnt-signaling pathway, as well as the vesicular transport signaling pathway (45–52). Importantly, large-scale identification of PARylation targets through transcriptomic and proteomic analyses revealed that these PARPs modulate RNA metabolism through the modification of RNA binding proteins involved in various levels of post-transcriptional regulation (53–55).

Recently, several reports have shown that PARylation activity is linked to the outcome of several muscle-related diseases including cancer-induced muscle wasting (cachexia), dystrophy, and sarcopenia (49,56–61). This is due, in part, to changes in mitochondrial biogenesis and production of pro-inflammatory cytokines (such as IL-6 and TNFα) (49,56,57,62–64). While these and other observations suggest that PARylation could play an important role in the physiology of skeletal muscle tissue and its ability to adapt to internal and external assaults, this possibility has not been fully explored. Indeed, although synthesis of PAR was suggested to be correlated with the differentiation of limb mesodermal cells into muscle cells (65), we do not know the PARPs involved in this process and whether/how PARylation could impact myogenesis, a process that drives muscle fiber formation during development as well as in response to injuries (66). Myogenesis is a multi-stage process through which mono-nucleated muscle precursor cells, called myoblasts, fuse to form multi-nucleated myotubes. This process is mediated through the controlled expression of pro- and anti-myogenic factors, including nucleophosmin (NPM) and myogenin, that collaborate together to ensure the commitment of myoblasts to the myogenic program (66–68). Therefore, uncovering the mechanisms by which PARylation impacts muscle fiber formation is an essential step toward our understanding of the physiological role of this important PTM.

In this study, we provide strong evidence that TNKS1-dependent PARylation is required for proper muscle fiber formation. TNKS1 achieves this effect by targeting promyogenic RNA binding proteins (RBPs), such as HuR, to modulate post-transcriptional events involved in the expression of key myogenic regulatory factors. HuR harbors a conserved TNKS1-binding motif that is essential for its PARylation as well as its promyogenic function. Together, our data uncover that TNKS1-mediated PARylation of RBPs could be a general mechanism required for proper muscle fiber formation.

## Materials and methods

### Cell culture

C2C12 muscle cells (ATCC, Manassas, VA, USA) were grown and maintained in 20% fetal bovine serum (FBS) (Sigma) and 1% penicillin/streptomycin antibiotics in DMEM (Dulbecco's modified Eagle's medium) (ThermoFisher). Primary myoblasts were harvested from murine hindlimb muscles (69) and were cultured in satellite cell media [DMEM glutamax (ThermoFischer), F12, 10% FBS, 1% UltroserG (Pall Life Sciences)]. Cells were grown in a humidified incubator at 37°C, 5% CO_2_. To induce muscle differentiation, both C2C12 and primary cells were switched to differentiation media (DMEM containing 2% horse serum (ThermoFisher) and 1% penicillin/streptomycin antibiotics) when they reached 100% confluency. Primary cells were plated in dishes coated with 0.1% gelatin in sterile water.

### XAV939 and PDD00017273 Treatment

C2C12 cells were treated with 10 μM XAV939 (Cedarlane), 5 uM PDD00017273 (Sigma-Aldrich) or DMSO as a negative control during the exponential phase and, additionally, upon induction of muscle cell differentiation.

### Plasmids and GST-tagged protein expression

The pGEX-6P1 plasmids containing the full length HuR were generated as previously described (70). The GST-HuR^G224D^ plasmid was generated by Norclone Biotech Laboratories. BL21 bacteria were transformed with either GST or the GST-HuR constructs described above. The expression of the proteins was induced by IPTG (0.5 mM for 4 h at 37°C) in a 1-l culture. The bacteria were collected and lysed. The GST proteins were pulled down using Glutathione Sepharose beads.

### Transfection

Transfections with siRNAs were performed when cells reached 50–60% confluency using Jetprime (Polyplus Transfection) for 48 h according to the manufacturer's instructions. For DNA plasmid, cells were transfected at 70% confluency with 1 μg/ml of plasmid DNA for 24 h using Jetprime as well. Cells were then switched to differentiation media when 100% confluency is reached and collected as indicated at various time points after the induction of differentiation. siRNAs used are listed in Supplementary Table S3. The GFP and GFP-HuR plasmids were generated as described in (71), while the GFP-HuR^G224D^ construct was generated by Norclone Biotech Laboratories. For the rescue experiments, subconfluent C2C12 cells were transfected with siRNA against HuR which specifically targets the 3′UTR of the mRNA to avoid targeting of the exogenous GFP-HuR constructs that were transfected the next day. On the following day, a second hit of transfection was performed for 4 hours, followed by a transfection using GFP, GFP-HuR and GFP-HuR^G224D^ plasmids. siRNAs used in the experiments were purchased from ThermoFisher unless stated otherwise (Supplementary Table S3).

### Immunofluorescence

Cells were fixed in 3% paraformaldehyde (Sigma) for 20 min. They were then permeabilized with a solution containing 0.5% Triton X-100 and 1% goat serum in phosphate-buffered saline (PBS) with agitation for 15 min. After washing with 1% goat serum in PBS, cells were incubated with primary antibodies against myosin heavy chain (MF-20, developmental studies Hybridoma Bank, 1:250), myoglobin (Abcam, 1:500) diluted in 1% goat serum in PBS for one hour at room temperature. Following further washing, cells were incubated with appropriately labeled Alexa Fluor^®^ (Invitrogen) secondary antibodies (1:1000) for an additional hour at room temperature. 4′,6-diamidino-2-phenylindole (DAPI) staining was used to visualize nuclei. Cells were visualized using a Zeiss Axio Observer.Z1 inverted microscope with a 63× oil objective, and images were obtained using an AxioCam MRm digital camera.

### Fusion index

The fusion index was used to determine the efficiency of C2C12 differentiation. It was quantified by calculating the ratio of the number of nuclei in myotubes versus the total number of nuclei counted in the same field. Fusion Index was quantified with Fiji (72) by analyzing 20× Immunofluorescence images (26 mm^2^ sample area). Total nuclei, 30–500 μm^2^ particles, were identified on the DAPI stain channel, creating a binary mask. A second mask of fibers based on the Myoglobin stain allowed the overlay of nuclei mask to fiber mask and identified fused against non-fused. Each sample (*n* = 3) was imaged in three different non-overlapping areas, and the fusion index was averaged for statistical analysis.

### Protein extraction and immunoblotting

Total cell extracts were prepared by lysing cells with mammalian lysis buffer (50 mM HEPES pH 7.0, 150 mM NaCl, 10% glycerol, 1% Triton, 10 mM pyrophosphate sodium, 100 mM NaF, 1 mM EGTA, 1.5 mM MgCl_2_, 1× protease inhibitor (Roche) and 0.1 M orthovanadate) for 15 min on ice, with vortexing every 5 min. Lysates were collected after centrifugation for 15 min at 12 000 rpm. The lysates were run on SDS-PAGE and transferred on nitrocellulose membranes (Bio-Rad), and then analyzed by western blotting using antibodies against HuR (3A2), pADPr (96, 10), TNKS1, KSRP, myogenin and NPM. Quantifications were performed using ImageJ and normalized to tubulin. Antibodies used are listed in Supplementary Table S3.

### Subcellular fractionation

siRNA-treated C2C12 cells were collected 2 days post-induction of differentiation. After washing with PBS, cells were resuspended in 500 μl EBKL buffer (25 mM HEPES, pH 7.6, 5 mM MgCl2, 5 mM KCl, 0.5% NP-40) and incubated 15 min on ice. Cells were then lysed on ice by 50 strokes in a Dounce-type homogenizer using the tight pestle. The homogenate was subject to a series of low-speed centrifugations to separate the cytoplasmic fraction (supernatant) from the nuclear fraction (pellet). Laemmli sample buffer was added to the samples and used for western blot experiments.

### *In vitro* PARylation assays

The PARP Universal Chemiluminescent assay kit (Trevigen #4676-096K) was used to test the PARylation of recombinant HuR by PARP1 (Enzo Life Sciences Enzo Life Sciences # ALX-201–063-C020), PARP3 (Enzo Life Sciences Enzo Life Sciences # 201-170-C020), and TNKS1 (BPS Bioscience # 80504) (Figure 2F, G). The TNKS1 Histone Ribosylation assay kit (Biotin-labeled NAD+) (BPS Bioscience # 80573) was used to compare the TNKS1-mediated PARylation of recombinant HuR and HuR^G224D^, since this kit is optimized for TNKS1-mediated PARylation. The assays were performed as per the manufacturer's protocol, except that 1.5 ml tubes were used rather than the 96-well plate provided. Briefly, PARP enzymes, biotinylated NAD+ and the PARP buffer were incubated with GST or the GST-HuR isoforms for 1 h at room temperature. GST-Sepharose beads (GE Healthcare) were added to the tubes, which were rotated for 30 min. The samples were washed with PBS. HRP-conjugated Streptavidin provided in the kits was added to the beads, which were rotated for 40 min at room temperature. The beads were washed again and transferred to 96-well plates where luminol (ECL) was added for chemiluminescence measurement by a Synergy Mx Multimode Plate Reader using the Gen5 Data Analysis software. Catalog numbers for the antibodies used in the study, as well as the company from which they were purchased, is shown in Supplementary Table S3.

### RNA-immunoprecipitation

Cells were lysed in lysis buffer (50 mM Tris–HCl pH 8.0, 0.5% triton 100×, 150 mM NaCl, 100 mM NaF, 1× protease inhibitors (Roche)). Pre-washed Protein A beads were incubated with the antibodies for 4 h, rotating at 4°C. The beads were washed three times with low salt buffer (50 mM Tris pH 8, 0.5% Triton X-100, 150 mM NaCl, 1× protease inhibitor). 800 ug of total cell extracts were added, and the samples were rotated overnight at 4°C. The next day, samples were washed three times with low salt buffer, and the co-immunoprecipitated RNA was purified and resuspended in 10 μl of nuclease-free water. 4 μl of the RNA was used for RT-qPCR analysis.

### Immunoprecipitation

Lysates were incubated with 5 μg antibodies overnight, rotating at 4°C. The following day, protein A/G magnetic beads (GE healthcare – 17152104011150) were added to the lysates and rotated for an hour at room temperature. The beads were washed three times with washing buffer (25 mM Tris–HCl pH 8.0, 650 mM NaCl, 0.05% Tween-20, 100 mM NaF, 1× protease inhibitors) by placing the tubes in the magnetic stand and removing the washing buffer. The beads were washed once with water. Laemmlli dye was added to the tubes, and the tubes were rotated for 10 min. The supernatant was analyzed by western blot.

### Actinomycin D pulse-chase experiments

Cells were transfected with scrambled control or siRNAs against TNKS1 or HuR. Two days after induction of differentiation, the cells were treated for 0, 1, 3 and 6 h with 2.5ug/ml of the RNA polymerase II inhibitor, actinomycin D (Act. D) (Sigma – A1410) to assess the stability of *NPM* and *myogenin*, mRNAs. RNA was extracted using Trizol reagent (Invitrogen) following the manufacturer's protocol. The level of *myogenin* and *NPM* mRNAs was determined by RT-qPCR and normalized to *Gapdh* mRNA levels in each sample. The stability was assessed by plotting the mRNA levels relative to the abundance of the messages at 0h of Act. D treatment, considered as 100%.

### Quantitative RT-PCR

One microgram of total RNA or four microliters of immunoprecipitated RNA was reverse transcribed using the 5X iScript reagent (Bio-Rad) according to the manufacturer's protocol. qPCR was done using 20-fold dilutions of the cDNA using SsoFast EvaGreen Supermix (Bio-Rad). RNA levels of the genes of interest were normalized to Gapdh mRNA levels by calculating the 2^−ΔΔ^*^C^*_T_ values, in which ΔΔC_T_ is the difference in C_T_ between the gene of interest and the housekeeping gene (Gapdh). The sequences of primers used in the experiments are shown in Supplementary Table S3.

### Mass spectrometry

*Sample preparation*: Following immunoprecipitation, pellets were washed three times with PBS. The pellets were then sent to Southern Alberta Mass Spectrometry Facility for preparation and analysis by mass spectrometry. 583 proteins were identified by selecting for unique count peptides, which are peptides that are identified in unique samples and not in all samples as they are considered background. By eliminating the proteins that were bound to the IgG control, 204 proteins remained (Supplementary Table S1). The list of proteins was subjected to PANTHER classification system (http://www.pantherdb.org/), selecting for classification by the Gene Ontology Molecular Function type of characterization.

### Statistical analyses

All values are reported as mean ± standard error of the mean (S.E.M.). The significance of the difference between the two-group means was assessed by unpaired *t*-test for normally distributed variables. The significance of differences between more than two group means was assessed by ANOVA test followed by Tukey HSD test. Tukey tests for one-way ANOVA were assessed by astatsa.com, and Tukey tests for two-way ANOVA were assessed by GraphPad Prism. *P*-values equal to or <0.05 were considered significant: 0.05–0.01 (*), 0.01–0.001 (**) and <0.001 (***).

## Results

### TNKS1-dependent PARylation activity is required for myogenesis

**Figure 1.**
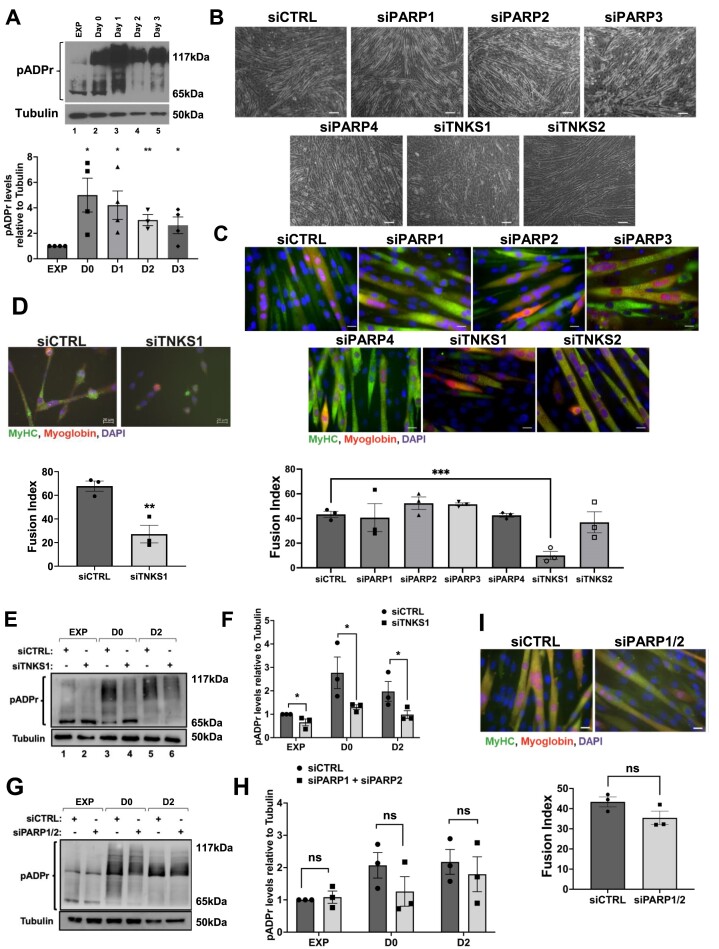
TNKS1 is required for muscle cell differentiation**. (A**) Total cell extracts were prepared from exponential and differentiating C2C12 myoblasts (Exp, Day 0, Day 1, Day 2, Day 3). (Top panel) Western blot experiments were performed using antibodies against pADPr or α-tubulin (loading control). (Bottom panel) Histogram representation of the quantification of the western blot in top panel. Values were quantified using ImageJ and normalized to tubulin. (**B**) Phase contrast images were taken of C2C12 cells treated with scrambled control (siCTRL), or specific siRNAs against PARP1, PARP2, PARP3, PARP4, TNKS1 or TNKS2 three days after induction of differentiation. Images of a single field are shown and represent three independent experiments (scale bars, 100 μm). (**C**) Immunofluorescence experiments demonstrating the differentiation of C2C12 myoblasts treated with the siRNAs described in (B). (Top panel) Cells were fixed three days post-induction of differentiation, and antibodies against known markers of muscle fibers (anti-MyHC, anti-myoglobin) were used for immunostaining. DAPI was used to stain nuclei. Images of a single field are shown and represent three independent experiments (scale bars, 20 μm). (Bottom panel) Quantification of the fusion index for the C2C12 cells described in top panel. The fusion index was calculated as the ratio of the nuclei number in myotubes versus the total number of nuclei. (**D**) (Top panel) Immunofluorescence experiments demonstrating the differentiation of primary myoblasts treated with siCTRL or siTNKS1. Cells were fixed 3 days post-induction of differentiation, and antibodies against known markers of muscle fibers (anti-MyHC, anti-myoglobin) were used for immunostaining. DAPI was used to stain nuclei. Images of a single field are shown and represent three independent experiments (scale bars, 20 μm). (Bottom panel) Quantification of the fusion index for the C2C12 cells described in top panel. The fusion index was calculated as the ratio of the nuclei number in myotubes versus the total number of nuclei. (E, F) Total extracts were prepared from C2C12 cells transfected with scrambled control (siCTRL) or siRNA against TNKS1 (siTNKS1) and collected from exponentially growing (EXP) and differentiating C2C12 myoblasts (Day 0 and Day 2). (**E**) The extracts were used in western blot analysis using antibodies against pADPr or α-tubulin (loading control). (**F**) Histogram representation of the quantification of the western blot in the left panel. Values were quantified using ImageJ, normalized to tubulin, and shown relative to the EXP siCTRL treated condition. (G, H) Total extracts were prepared from C2C12 cells transfected with scrambled control (siCTRL) or a combination of siRNA targeting PARP1 and PARP2 (siPARP1/2) and collected from exponentially growing (EXP) and differentiating C2C12 myoblasts (Day 0 and Day 2). (**G**) The extracts were subjected to western blot analysis using antibodies against pADPr or α-tubulin (loading control). (**H**) Histogram representation of the quantification of the western blot in the left panel. Values were quantified using ImageJ, normalized to tubulin, and shown relative to the EXP siCTRL treated condition. (**I**) (Top panel) Immunofluorescence images of C2C12 transfected with scrambled control (siCTRL) or a combination of siRNA against PARP1 and PARP2 (siPARP1/2) Cells were fixed three days post-induction of differentiation, and antibodies against known markers of muscle fibers (anti-MyHC, anti-myoglobin) were used for immunostaining. DAPI was used to stain nuclei. Images of a single field are shown and represent three independent experiments (scale bars, 20 μm). (Bottom panel) Quantification of the fusion index for the C2C12 cells in top panel. The fusion index was calculated as the ratio of the nuclei number in myotubes versus the total number of nuclei. Data shown in Figure 1 are presented ± the s.e.m. of three independent experiments with *P < 0.05, **P < 0.01, ***P < 0.001 by unpaired *t*-test.

In order to determine the role of PARPs during myogenesis, we assessed, as a first step, PARylation activity during the differentiation of C2C12 muscle cells (73) (Supplementary Figure S1). Using western blot experiments with an anti-pADPr antibody, which is one of the gold standard method used to identify PARylated proteins in extracts (15), we observed a significant increase of PARylation activity in cells that are ready to be differentiated into myotubes (Figure 1A). This level of PARylation persisted during the differentiation process. To investigate the importance of PARylation during myogenesis, we used two different sets of siRNAs to individually deplete PARP enzymes (PARP1, PARP2, TNKS1 or TNKS2) in C2C12 cells that were subsequently induced for differentiation. The impact of depleting PARP3 and PARP4, which modify proteins through mono(ADP-ribosylation) rather than PARylation (9,10) was included as controls. Each set of siRNAs was equally efficient in knocking down the PARPs in C2C12 myoblast cells (Supplementary Figure S2). Our results show, using the two different sets of siRNAs, that the depletion of TNKS1, but not the other PARPs, prevented the commitment of C2C12 cells to the myogenic process (Figure 1b-c, Supplementary Figure S3a, b). Of note, although TNKS2 has redundant functions with TNKS1 (35), its depletion did not affect the formation of myotubes (Figure 1B, C, Supplementary Figure S3a, b). Importantly, we also further confirmed that the knockdown of TNKS1 prevented the differentiation of murine primary myoblasts isolated from mice (Figure 1D, Supplementary Figure S3c). We next determined if TNKS1 is responsible for the increase in the PARylation activity observed during myogenesis (Figure 1a). Towards this end we knocked down TNKS1 and evaluated the levels of PARylated proteins during myogenesis. In the absence of TNKS1, the level of PARylated proteins in differentiating muscle cells was reduced by ∼2-fold (Figure 1E, F). We further showed that the involvement of PARPs in the catalysis of PAR upon induction of muscle differentiation is limited to the activity of TNKS1 since the dual knockdown of PARP1 and PARP2 (the two main PARPs with redundant roles in the generation of PAR(25)) did not affect the total levels of PAR observed during muscle cell differentiation (Figure 1G, H). This result is in line with the observation that the simultaneous knockdown of both PARP1 and 2 (similarly to what was observed for each PARP individually in Figure 1b-c) did not affect the differentiation of C2C12 cells (Figure 1I). The importance of TNKS1 in this process was further confirmed using the TNKS1 inhibitor XAV939 (52,74,75). XAV939 but not DMSO (used as a negative control) significantly decreased the levels of PARylated proteins during myogenesis as well as reduced the formation of myotubes by ∼40% (Supplementary Figure S4).

The possibility exists that the effect of knocking down or inhibiting TNKS1 on muscle differentiation could result from an indirect effect on the expression of PARP1 or TNK2. In order to rule out this possibility, we assessed the effect of knocking down TNKS1 on the expression of these other PARPs. We observed that while the depletion of TNKS1 had no effect on TNK2 expression in myotubes, it did, to our surprise, significantly increase by more than twofold PARP1 protein levels (Supplementary Figure S5a, b). This indicates that the effect of knocking down TNKS1 on the myogenic process is not due to the decreased expression of PARP1 or TNKS2. These results, coupled with the fact that the expression of endogenous PARP1 (but not TNK1) decreased during muscle cell differentiation (Supplementary Figure S5c, d), therefore indicate that TNKS1-dependent PARylation is required for the formation of muscle fibers.

### TNKS1 promotes myogenesis by PARylating key promyogenic RNA-binding proteins

**Figure 2.**
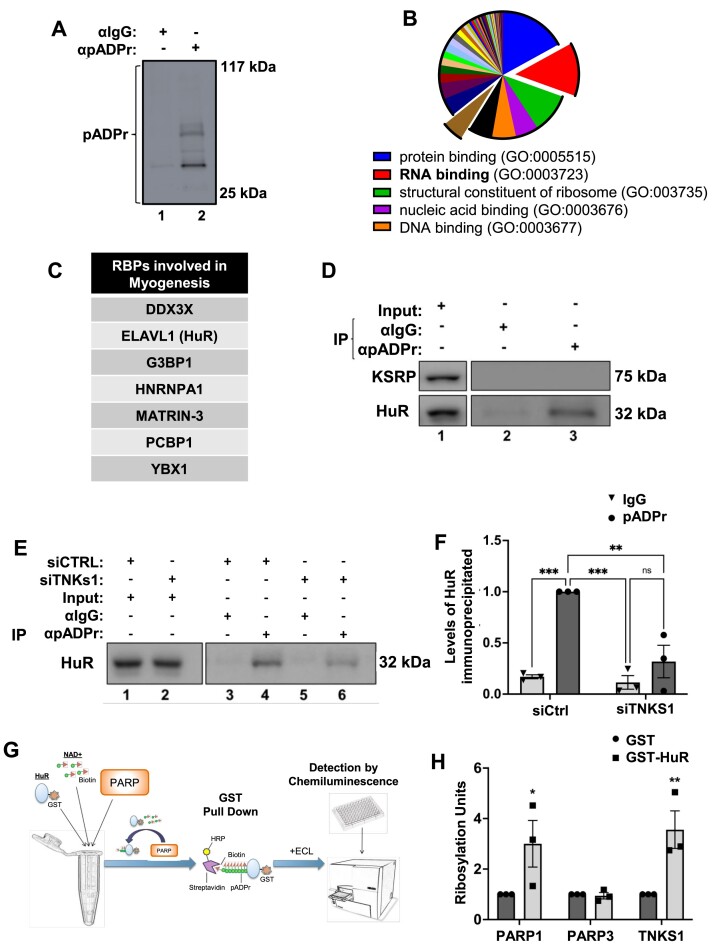
TNKS1 PARylates promyogenic RNA-Binding Proteins such as HuR. (**A**) Total cell extracts isolated from differentiating C2C12 myoblasts (Day 2) were used for immunoprecipitation experiments with pADPr or IgG antibodies and analyzed by mass spectrometry. The immunoprecipitation of pADPr was validated by western blot analysis using a pADPr antibody. The blot shown is representative of three independent experiments. (**B**) The identified proteins were classified according to their gene ontology molecular function. The percentage of proteins identified in each category is represented in a pie chart. (Lower panel) List of top 5 categories. (**C**) List of RNA-binding proteins previously associated with myogenesis identified in (B). (**D**) Immunoprecipitation experiments using pADPr or IgG antibodies were performed with extracts from confluent (D0) C2C12 cells. The association of RNA-binding proteins such as HuR and KSRP to pADPr was determined by western blot analysis. The blot shown is representative of three independent experiments. (E, F) Immunoprecipitation experiments using pADPr or IgG antibodies were performed with extracts from confluent (D0) C2C12 cell transfected with scrambled control (siCTRL) or siRNA against TNKS1 (siTNKS1). (**E**) The association of HuR to pADPr was determined by western blot analysis. The blot shown is representative of three independent experiments. (**F**) Histogram representation of the quantification of the western blot in the (E). (**G**) Schematic representation of the *in vitro* PARylation assay procedure. GST-HuR and biotinylated NAD+ were incubated with recombinant PARP1, PARP3, or TNKS1 enzymes. HRP-conjugated streptavidin was added to the reaction and the signal was measured as arbitrary units by chemiluminescence by detection with luminol. (**H**) Quantification of Ribosylation units demonstrating the PARylation of GST-HuR by PARP1 and TNKS1, but not PARP3, *in vitro*. Levels are normalized to GST control. Data shown in Figure 2 are presented ± the s.e.m. of three independent experiments with *P < 0.05, **P < 0.01, ***P < 0.001 by unpaired *t*-test

To decipher the mechanism through which TNKS1 regulates myogenesis, we began by identifying the network of proteins that are PARylated by this enzyme in muscle fibers. To this end, we performed protein identification by mass spectrometry analysis on pellets obtained from an immunoprecipitation experiment using anti-pADPr or anti-IgG (negative control) antibodies and lysates from differentiated C2C12 myotubes (Figure 2A). We identified 204 proteins specifically associated with affinity-purified PAR (Supplementary Table S1). Classification of these proteins, based on known molecular function, using the Panther software, revealed that a predominant group of 87 proteins belong to the family of RNA binding proteins (RBPs) (Figure 2B and Supplementary Table S2). Among these, 7 proteins (Figure 2C) have been previously associated with muscle function/integrity (66,76–81). From this short list, HuR (ELAVL1) is the only RBP that has been extensively characterized as one of the key post-transcriptional regulators of muscle fiber formation and function both *in vitro* and *in vivo* (82–86). In addition HuR has been identified as both a non-covalent PAR reader (87,88) and a covalently PARylated protein in proteome-wide analysis of PAR-associated protein (54,89). Therefore, as a proof of concept for the role of PARylation in muscle fiber formation, in this study, we chose to delineate the role of TNKS1 in the promyogenic function of HuR. First, we confirmed the PARylation of HuR during myogenesis by repeating the immunoprecipitation experiment described above (with anti-pADPr or anti-IgG antibodies) followed by western blot analysis using anti-HuR and -KSRP antibodies (27,90). We included KSRP as a negative control since it was not found in our list of RBPs that are PARylated in muscle fibers (Supplementary Table S2). We observed that anti-pADPr antibody immunoprecipitated HuR but not KSRP (Figure 2D). Moreover, TNKS1 knockdown significantly reduced the level of PARylated HuR (Figure 2E, F). Of note, since the level of HuR in muscle cells was not affected by the absence of TNKS1 (Supplementary Figure S6a), we concluded that the observed decrease in pADPr-HuR association is not due to an effect on HuR expression.

While these results clearly indicate that, in differentiating muscle cells, TNKS1 is responsible for HuR PARylation, they do not provide any info on whether this effect is due to a direct or indirect interaction PARylation of HuR by TNKS1. To address this, we performed an *in vitro* PARylation assay (Figure 2G) (91) using recombinant TNKS1 and HuR. PARP1 and PARP3 were also included in the assay as a positive and negative control respectively since PARP1 was previously shown to PARylate HuR in macrophage cells (91) while PARP3 is a MART (92). Similarly, to PARP1, TNKS1 but not PARP3 PARylated HuR *in vitro* (Figure 2H). Additionally, XAV939 drastically reduced HuR PARylation in differentiated muscle cells (Supplementary Figure S6b). Together, these results suggest that, during muscle cell differentiation, TNKS1 is the main PARP responsible for the PARylation of promyogenic RBPs such as HuR.

### TNKS1-mediated PARylation is required for HuR function during myogenesis

**Figure 3.**
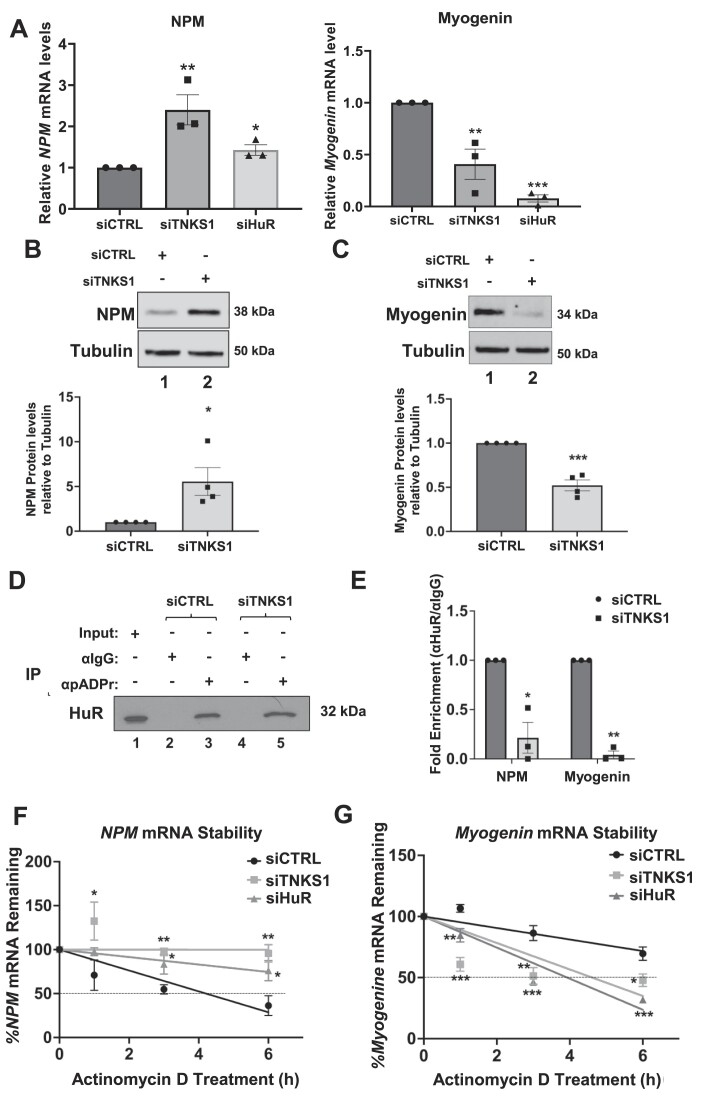
TNKS1-mediates binding of HuR to myogenic mRNA targets during myogenesis. C2C12 muscle cells were transfected with scramble control (siCTRL) or siRNAs specific for TNKS1, or HuR. Total RNA and protein lysates were prepared from these cells 2 days post-induction of differentiation. (**A**) *NPM* (left panel) and *Myogenin* (right panel) mRNA levels were determined by RT-qPCR, standardized against *GAPDH* mRNA, and expressed relative to siCTRL conditions. (B, C) Total extracts were used for western blot analysis to determine NPM (**B**) and Myogenin (**C**) protein levels. (Bottom) Histogram representation of the quantification of the western blot. Values were quantified using ImageJ, normalized to tubulin, and shown relative to the siCTRL treated condition. (D, E) RNA-Immunoprecipitation coupled to RT-qPCR experiments was performed using anti-HuR and anti-IgG antibodies on total extracts from differentiating C2C12 cells treated with scrambled control (siCTRL) or siRNA against TNKS1 (siTNKS1). (**D**) Western blot assessing the immunoprecipitation of HuR. (**E**) *NPM* and *Myogenin* mRNA levels in the immunoprecipitates were normalized to the corresponding IgG sample and mRNA input. The levels of *NPM* and *myogenin* mRNA in siTNKS1 conditions were plotted relative to siCTRL conditions. (F, G) Actinomycin D (Act. D) pulse-chase assays were performed using C2C12 myoblasts transfected with scrambled control (siCTRL) or siRNA against TNKS1 (siTNKS1). 48 h post-induction of differentiation, the cells were treated for various periods of time with Actinomycin D to assess the stability of *NPM* (**F**) and *Myogenin* (**G**) mRNAs. Data shown in Figure 3 are presented ± the s.e.m. of three independent experiments with *P < 0.05, **P < 0.01, ***P < 0.001 by unpaired *t*-test.

One of the main features of the promyogenic function of HuR is its functional dichotomy. Indeed, others and we have demonstrated that, to promote myogenesis, HuR simultaneously exercises two opposite functions on some of its target mRNAs: the decay of nucleophosmin (*NPM*) and the stability of *myogenin* (66,83,85,93). Therefore, we investigated the impact of TNKS1-mediated PARylation on these two opposite but complementary functions of HuR during myogenesis. Our data show that the depletion of TNKS1 in C2C12 cells (using our two different sets of siRNAs), similarly to what was observed for the knockdown of HuR (83,85,94), differentially impacted the expression levels of NPM (increase) and *myogenin* (decrease) mRNAs and proteins (Figure 3A–C, Supplementary Figure S7a–c). Next, we performed immunoprecipitation experiments with the anti-HuR antibody on extracts from differentiating C2C12 cells depleted or not of TNKS1, and the association of *NPM and myogenin* mRNAs was assessed by RT-qPCR analysis. Our data show that TNKS1 depletion significantly reduced (by >3-fold) the association of HuR to both *NPM* and *myogenin* mRNAs (Figure 3D, E, Supplementary Figure S7d, e). The effect of TNKS1 depletion on NPM and myogenin expression as well as their binding to HuR were also confirmed using XAV939 (Supplementary Figure S8). Next, actinomycin D pulse-chase experiments (83,93,94) were used to determine the impact of TNKS1 on the half-lives of *NPM* and *myogenin* mRNAs. The knockdown of TNKS1 in C2C12 cells, similarly to HuR depletion (83,85,94), increased the half-life of *NPM* mRNA, while at the same significantly decreased the stability of *myogenin* mRNA (Figure 3F, G, Supplementary Figure S7f, g). Therefore, together, these observations clearly establish that the TNKS1-mediated PARylation of RBPs of HuR is required for the post-transcriptional regulation of key promyogenic factors such as NPM and Myogenin.

**Figure 4.**
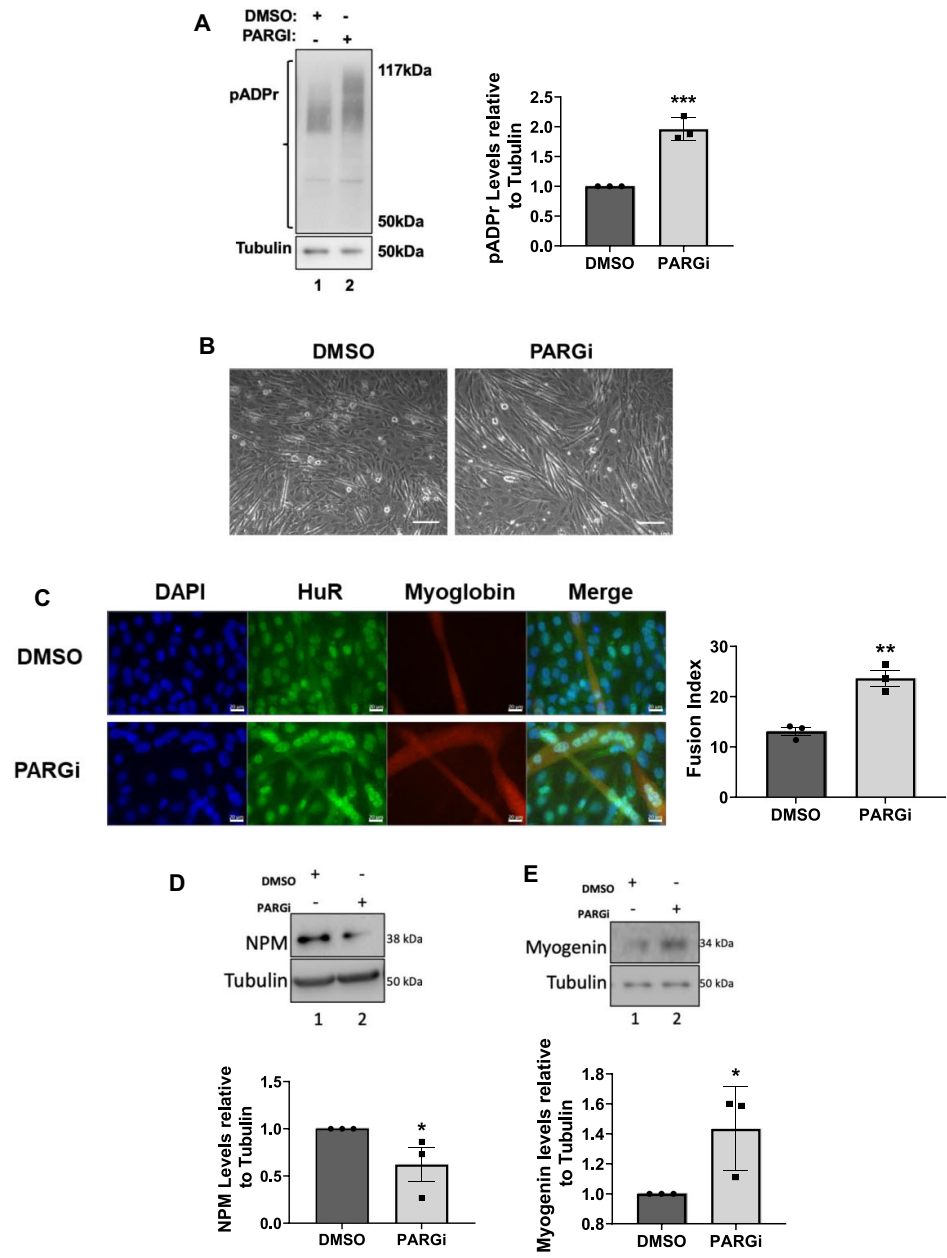
PARG inhibition ameliorates muscle fiber formation. (**A**) (Left panel) Total cell extracts were prepared from differentiating C2C12 cells (Day 2) treated with 5uM PARGi or DMSO as a control. These extracts were used for western blot analysis with antibodies against pADPr or α-tubulin (loading control) (Right panel). Histogram representation of the quantification of the western blot Levels of PARylated proteins were quantified using ImageJ and normalized to tubulin and shown relative to DMSO treated control condition. (**B**) Phase contrast images were taken of differentiating C2C12 cells treated with 5 uM PARGi or DMSO (Day 2). Images of a single field are shown and represent three independent experiments (scale bars, 50 μm). (**C**) (Left panel) Immunofluorescence experiments on differentiating C2C12 myoblasts treated with 5 uM PARGi or DMSO. Staining was performed with antibodies against known markers of muscle fiber formation, MyHC and myoglobin. DAPI was used to stain nuclei. Images of a single field are shown and represent three independent experiments (scale bars, 20 μm). (Right panel) Quantification of the fusion index for the C2C12 cells described in the left panel. The fusion index was calculated as the ratio of the nuclei number in myotubes with two or more nuclei versus the total number of nuclei. (D, E) (Top panels) Total cell extracts were prepared from differentiating C2C12 cells (Day 2) treated with 5 uM PARGi or DMSO as a control and used for western blot analysis with antibodies against NPM (**D**), Myogenin (**E**) or α-tubulin (loading control). (Bottom panels) Histogram representation of the quantification of the western blots on top panels. Values were quantified using ImageJ, normalized to tubulin, and shown relative to the DMSO-treated control condition. Data shown are presented ± the s.e.m. of three independent experiments with *P < 0.05, **P < 0.01, ***P < 0.001 by unpaired *t*-test.

To confirm the importance of TNKS1-mediated PARylation in differentiating muscle cells, we treated cells (induced for muscle differentiation) with a PARG inhibitor PDD00017273 (PARGi), which potently inhibits pADPr glycohydrolase (PARG), an enzyme that hydrolyzes pADPr, allowing the accumulation of pADPr in the cells (Figure 4A). We show, using PARGi, that inhibition of PARG activity increased the efficiency of muscle cell differentiation (Figure 4B, C), which is due, in part, to the decreased expression of NPM and increased expression of myogenin during this process (Figure 4D, E). Cumulatively, our results, therefore, show that PARylation represents a key PTM needed for the TNKS1-induced formation of myotubes.

**Figure 5.**
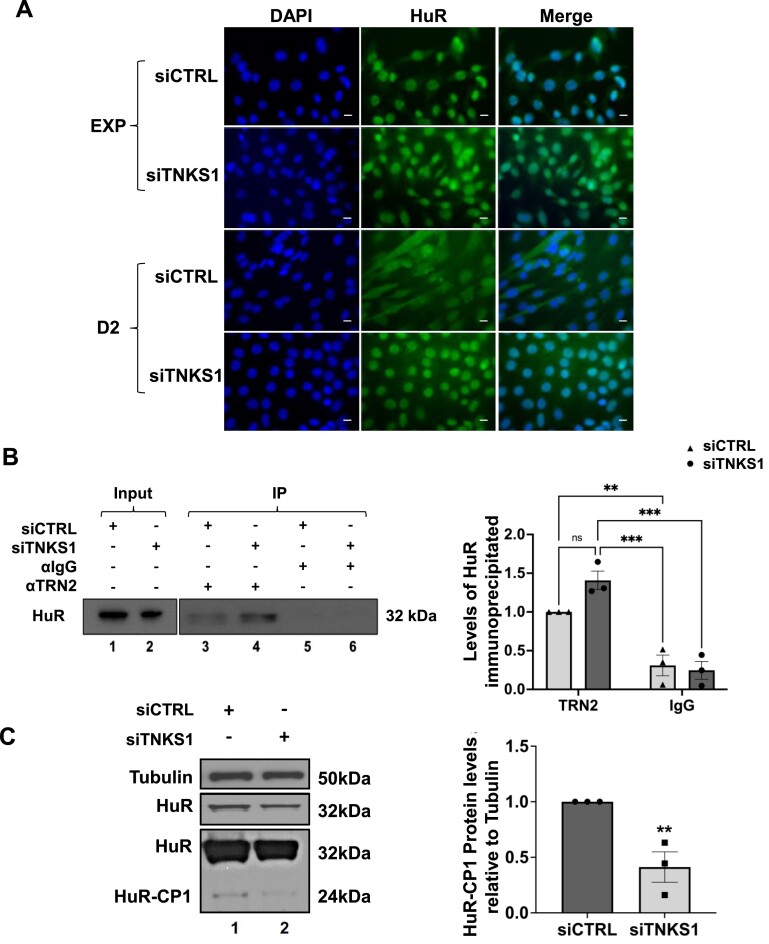
TNKS1-mediated PARylation of HuR regulates its cellular movement. (**A**) Immunofluorescence images showing the localization of HuR in exponentially growing (EXP) and differentiating (Day 2) muscle myoblasts transfected with scramble control (siCTRL) or siRNA against TNKS1 (siTNKS1). Immunofluorescence staining was performed with an antibody against HuR. DAPI was used to stain nuclei. Images of a single field are shown and are representative of three independent experiments (scale bars, 20 μm). (**B**) (Left panel) Immunoprecipitation experiments using TRN2 or IgG antibodies were performed with extracts from differentiating (D2) C2C12 cells transfected with scrambled control (siCTRL) or siRNA against TNKS1 (siTNKS1). The association of HuR to TRN2 was determined by western blot analysis. The blot shown is a representative of three independent experiments. (Right panel) Histogram representation of the quantification of the western blot in the left panel. (**C**) Total extracts from C2C12 myoblasts transfected at the exponential growth phase with scrambled control (siCTRL) or siRNA against TNKS1 (siTNKS1) and collected 2 days post-induction of differentiation, were used for western blot analysis (left panel) to determine HuR-CP1 protein levels. (Right panel) Histogram representation of the quantification of the western blot in left panel. Values were quantified using ImageJ and normalized to α-tubulin and shown relative to the siCTRL treated condition. Data shown in Figure 5 is presented ± the s.e.m. of three independent experiments with **P < 0.01, ***P < 0.001 by unpaired *t*-test.

We have previously shown that the cytoplasmic translocation of HuR is essential for its pro-myogenic function (66,82). In fact, during muscle cell differentiation, about 10–15% of HuR localizes to the cytoplasm where it is cleaved by caspase-3/7 generating two cleavage products: HuR-CP1 (24kD) and HuR-CP2 (8kD) (66,82). We also showed that HuR-CP1 competes with HuR for the binding to transportin-2 (TRN2), an import factor responsible of the movement of HuR from the cytoplasm to the nucleus (66,82). Consequentially, HuR-CP1 competes with the remaining non-cleaved HuR for its association with TRN2, causing the cytoplasmic accumulation of HuR (66,82). Therefore, as a next step, we determined the impact of depleting TNKS1 on the localization and the cleavage of HuR during myogenesis. Immunofluorescence experiments assessing the localization of HuR in differentiating muscle cells showed that, while as expected (93), HuR partially accumulates in the cytoplasm in control conditions, HuR is completely sequestered in the nucleus upon TNKS1 knockdown (Figure 5A, Supplementary Figure S9a). These results were validated biochemically by performing subcellular fractionation coupled to western blot experiments assessing the levels of HuR in the nucleus and cytoplasmic fractions of C2C12 myotubes depleted or not of TNK1 (Supplementary Figure S9b, c). Similarly, treating cells with XAV939 also resulted in the nuclear accumulation of HuR (Supplementary Figure S10). We then verified if this nuclear accumulation is the result of an inhibition of HuR export from the nucleus, or rather an increased import of HuR from the cytoplasm. Immunoprecipitation assays using anti-TRN2 or anti-IgG antibodies indicated that in muscle cells depleted of TNKS1, HuR associates more to TRN2, suggesting an increase in TRN2-mediated HuR import (Figure 5B). Additionally, the observed nuclear accumulation of HuR should in principle correlate with a decrease in its caspase-mediated cleavage. This was indeed the case since the depletion of TNKS1 in C2C12 resulted in a significant decrease in the levels of HuR-CP1 (Figure 5C). As such, our results show that TNKS1-mediated PARylation promotes the pro-myogenic function of HuR not only by enabling its interaction with target messages, but also by promoting its cytoplasmic accumulation and caspase-mediated cleavage.

### TNKS1 associates with HuR via a conserved Tankyrase-binding motif

**Figure 6.**
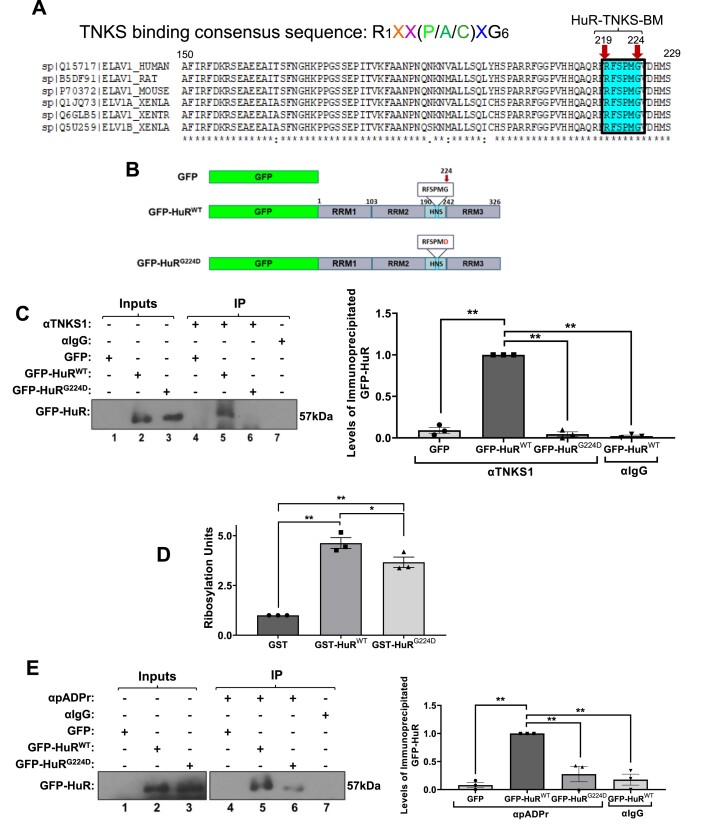
HuR contains a TNKS1 consensus binding motif. (**A**) Swiss-Prot Database (www.expasy.ch/prosite) blast of the putative TNKS1-binding motif identified in HuR. (**B**) Schematic of the GFP, GFP-HuR^WT,^ and GFP-HuR^G224D^ constructs. (**C**) (Left panel) Immunoprecipitation experiments using differentiating C2C12 cells expressing GFP, GFP- HuR^WT^ and GFP-HuR^G224D^ were performed using anti-IgG or anti-TNKS1 antibodies to assess the association of the exogenous proteins to TNKS1 by western blot analysis. The data are representative of three independent experiments. (Right panel) Histogram representation of the quantification of the western blot in the left panel. (**D**) *In vitro* PARylation assay: Quantification of Ribosylation units showing the PARylation of GST-HuR^WT^ and GST- HuR^G224D^ by recombinant TNKS1 *in vitro*. Levels are normalized to GST control. Data shown are presented ± the s.e.m. of three independent experiments with *P < 0.05, **P < 0.01 by one-way ANOVA, Tukey HSD test. (**E**) (Left panel) Immunoprecipitation experiments using differentiating C2C12 cells expressing GFP, GFP-HuR^WT^ or GFP- HuR^G224D^, were performed with anti-IgG and anti-pADPr antibodies to assess the PARylation by western blot analysis. The blot is representative of three independent experiments. (Right panel) Histogram representation of the quantification of the western blot in the left panel. Data shown in Figure 6 is presented ± the s.e.m. of three independent experiments with **P < 0.01 by unpaired *t*-test.

**Figure 7.**
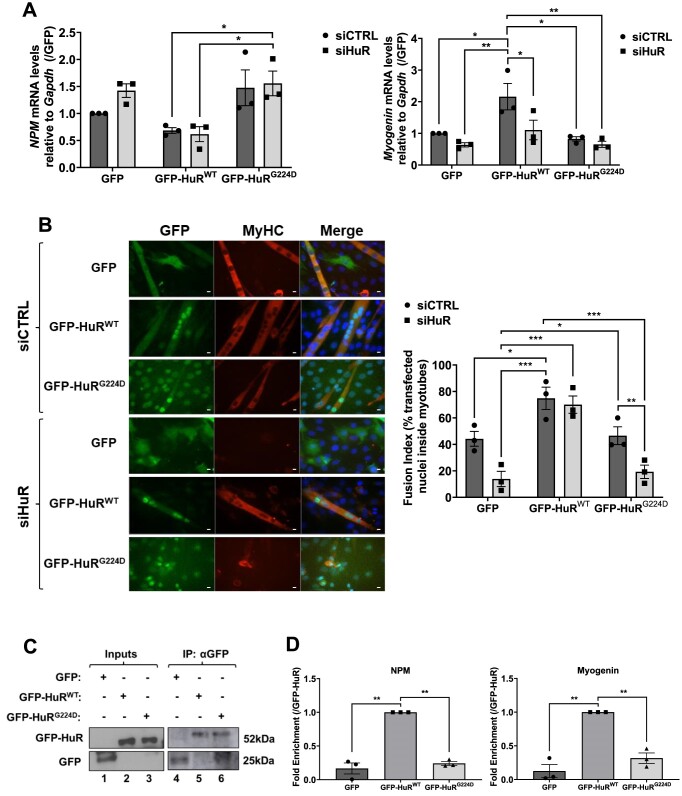
TNKS1-mediated PARylation of HuR is required for its pro-myogenic function. C2C12 cells were transfected with scrambled control (siCTRL) or siRNA against HuR (siHuR), and then constructs expressing GFP, GFP-HuR^WT^ or GFP-HuR^G224D^. (**A**) Total RNA was isolated from these C2C12 and the level of *NPM* (left graph) and *myogenin* (right graph) mRNAs was assessed by RT-qPCR, standardized against *GAPDH* mRNA, and expressed relative to siCtrl + GFP conditions. Data are represented as mean is presented ± the s.e.m. of three independent experiments with *P < 0.05, **P < 0.01 by two-way ANOVA, Tukey HSD test. (**B**) (Left Panel) Immunofluorescence (staining with anti-MyHC and anti-GFP antibodies, as well as with DAPI to stain nuclei) images of cells described in (A) assessing rescue of the myogenic phenotype in HuR knockdown cells (scale bars 20 μm). (Right panel) A histogram representation of the fusion index of immunofluorescence shown in left panel. Data represented as mean ± the s.e.m. of three independent experiments with *P < 0.05, **P < 0.01, ***P < 0.001 by two-way ANOVA, Tukey HSD test. (C, D) RNA-Immunoprecipitation experiments using differentiating C2C12 cells expressing GFP, GFP-HuR^WT^ and GFP-HuR^G224D^ were performed using anti-GFP antibodies. (**C**) Western blot confirming the immunoprecipitation of GFP-conjugated HuR isoforms. The blot is representative of three independent experiments. (**D**) Association of GFP, GFP-HuR^WT^ and GFP-HuR^G224D^ to *NPM* (left panel) *or myogenin* (right panel) was determined by RT-qPCR analysis. Data in Figure 7D are presented as the mean ± SEM (*n*= 3) with **P < 0.01 by one-way ANOVA, Tukey HSD test.

The TNKS1-mediated enzymatic activity requires the direct association of TNKS1 to its target proteins via a consensus motif known as the tankyrase binding motif (TBM) (95–98). TBM consists of six residues (RXXPXG), with arginine and glycine being the most critical for binding (95–99). Interestingly, scanning the primary sequence of HuR we identified a potential TBM in its C-terminal region (^219^RFSPMG^224^) (that we dub HuR-TBM) that is conserved across different species such as human, rat, mouse, and *xenopus* (Figure 6A). It was previously shown that mutating the glycine residue in the TBM of a given protein completely abolishes its ability to bind TNKS1 (99). Hence, to assess if the HuR-TBM is required for the interaction of HuR with TNKS1 in myotubes, we generated constructs expressing GFP-HuR wild-type (GFP-HuR^WT^) or GFP-HuR containing a glycine (G) → aspartate (D) mutation at the 224 position (GFP-HuR^G224D^) (Figure 6B). Our data show that the G→D mutation at the 224 residue abolished the interaction of HuR with TNKS1 (Figure 6C). Subsequently, we conducted an *in vitro* PARylation assay using GST-HuR^WT^ or GST-HuR^G224D^, and GST alone as a negative control to assess the importance of the HuR-TBM for the TNKS1-mediated PARylation of HuR (Figure 6D and Supplementary Figure S11). We observed a significant decrease in the PARylation levels of GST-HuR^G224D^ when compared to its wild-type counterpart (Figure 6D), suggesting that an intact HuR-TBM is required for the TNKS1-mediated PARylation of HuR *in vitro*. To determine the importance of this motif in HuR PARylation in myotubes, we performed immunoprecipitation experiments with anti-pADPr or anti-IgG antibodies on myotube extracts expressing the two HuR isoforms. Consistent with the above-mentioned results, the PARylation level of GFP-HuR^G224D^ was substantially reduced when compared to that of GFP-HuR^WT^ (Figure 6E). Rescue experiments in HuR-depleted muscle cells showed that GFP-HuR^WT^ but not its mutant counterpart, was able to re-establish the expression of HuR mRNA targets *NPM* and *myogenin* (Figure 7A) as well as the ability of these cells to enter myogenesis (Figure 7B and Supplementary Figure S12). In line with the results shown in Figure 5A and Supplementary Figure S9a–c, we show that mutation of the TBM affected the localization of HuR to the cytoplasm in differentiating muscle cells (Figure 7B). By performing RNA-immunoprecipitation experiments with anti-GFP antibody on extracts from cells expressing the two HuR isoforms, we next showed that these effects on myogenesis and the expression of *NPM* and *myogenin mRNAs* are due to the inability of GFP-HuR^G224D^ to associate with these messages (Figure 7C, D). Together, these observations demonstrate that HuR harbors a *bona fide* TBM that is required for its PARylation by TNKS1 and that this PTM is essential for its promyogenic function.

## Discussion

**Figure 8.**
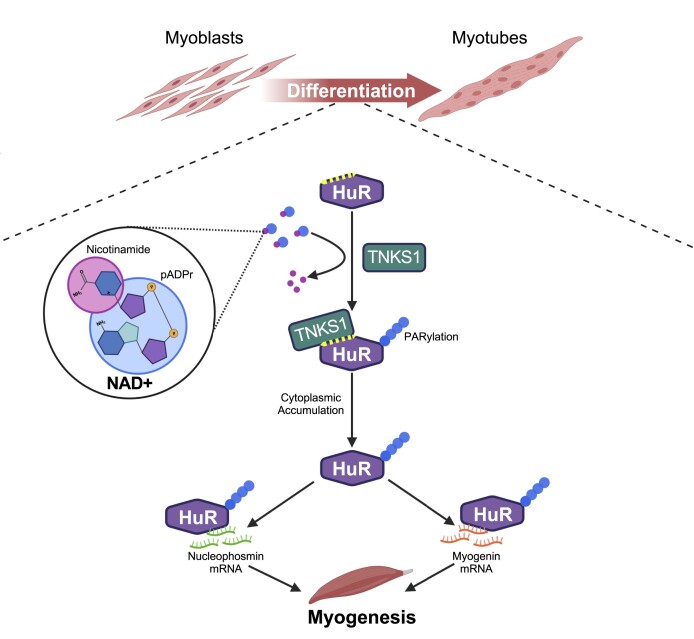
Model depicting the mechanism by which TNKS1-mediated PARylation of the promyogenic RBP HuR impact myogenesis. TNKS1-mediated PARylation promotes the cytoplasmic translocation of HuR. The observation reported in this study suggests that by retaining its PARylation status, HuR promotes myogenesis by differentially regulating the turnover and expression of its target mRNA *NPM* (decay) and *myogenin* (stability). Image was created with Biorender.com.

Although PARylation has been shown to be implicated in the onset of several skeletal muscle pathologies (56–59,62,100), its role in the induction of muscle cell differentiation remains elusive. In this study, we investigated the importance of this post-translational modification in the myogenic process. We showed that TNKS1-mediated PARylation is required for muscle cell differentiation. Our data demonstrate that TNKS1 is essential for this process since its depletion or chemical inhibition reduces muscle fiber formation. We show that these effects are due to TNKS1-mediated PARylation of the RBP HuR. TNKS1 modulates the cytoplasmic accumulation of HuR, as well as the binding of HuR to its mRNA targets, such as *NPM and myogenin*, resulting in the regulation of their turnover. We, furthermore, showed that TNKS1 binds HuR via a conserved consensus motif (HuR-TBM). Mutating the TBM of HuR prevented the interaction of HuR with target messages and the rescue of their expression in HuR-depleted conditions. More importantly, the mutant could not rescue the myogenic phenotype when overexpressed in cells depleted of HuR, a condition known to impair muscle fiber formation (84). Thus, our work reveals a new mechanism where the TNKS1-mediated PARylation of HuR is a key requisite for the induction of muscle cell differentiation (Figure 8).

Compounds that inhibit PARP1/2-mediated PARylation activity have been shown to ameliorate muscle performance/function in muscle-related diseases such as sarcopenia, Duchene muscle dystrophy, and cachexia (57–59,61,100,101). The onset of these diseases, therefore, seems to correlate with an increase in the activity of PARP1/2.

Our data indicate that PARP1 is not involved in promoting myogenesis and that it is rather TNKS1 that is involved in the process. Although both PARP1 and TNKS1 mediate PARylation of proteins, both have unique and specific roles in various cellular processes. Thus, the fate of muscle (whether it is formed or wasted) may depend on the specific PARP which is expressed and active under these conditions. We, as well as others, have shown that the expression of PARP1 decreases upon induction of muscle cell differentiation [Supplementary Figure S5c, d, (63,102,103)]. The decreased expression of PARP1 is thought to be a requisite for the induction of the myogenic process and the health of muscle fibers (63,103). Indeed, targeting the expression of PARP1 in myoblasts increases the expression of promyogenic factors as well as the increases their resistance to oxidative stress. Interestingly, our results suggest that TNKS1, in contrast to PARP1, beneficially modulates the formation of skeletal muscle. In agreement with this, TNKS1 is known to regulate the canonical Wnt signalling pathway which was previously shown to be involved in embryonic and adult skeletal muscle formation (50,52,104–110). Knockout mouse models of Wnt or Wnt signalling effectors display early embryonic lethality due to pronounced tissue damage and poor muscle development (111). During adult myogenesis or regeneration, Wnt1 has been shown to induce the expression of the MRF Myf5, whereas Wnt3 is involved in satellite cell differentiation (112,113). Additionally, both Wnt1 and Wnt3 are heavily involved in somitic myogenesis (114). Interestingly, others have also shown that XAV939 treatment decreases PARylation in rat L6 skeletal muscle cells (49). Therefore, the PARylation of RBPs such as HuR by TNKS1, in addition to the activation of the Wnt signalling pathway, may explain the importance of TNKS1 in modulating the myogenic process.

Post-translational modifications of HuR have been previously shown to play an important role in regulating its function. For example, phosphorylation of HuR by the G2-phase kinase Cdk1 on the Ser202 residue promotes the interaction of HuR with 14–3–3, resulting in its nuclear localization (115). The caspase-mediated cleavage of HuR on the D226 residue is another modification that modulates the localization of HuR during apoptosis and myogenesis leading respectively to the stabilization and expression of pro-apoptotic and pro-myogenic messages (71,82). Post-translational modifications can also impact the interaction of HuR with target messages. For instance, phosphorylation of HuR by the cell cycle checkpoint kinase Chk2 upon IR treatment led to a global decrease in HuR association to mRNA (116). More recently, HuR was shown to be modified by PARP1-mediated PARylation under inflammatory conditions and that this modification impacted the localization and the RNA-binding activity of HuR (91). Indeed, in activated macrophages, PARP1 PARylates HuR on the D226 residue and promotes its association to proinflammatory messages. Our work uncovers that PARylation is also important for the promyogenic function of HuR. However, it is TNKS1 but not PARP1 that is responsible for the PARylation of HuR during myogenesis. TNKS1-mediated PARylation of HuR promotes its mRNA binding ability and function during myogenesis. Additionally, in this work, we show that TNKS1-mediated PARylation is essential for the cytoplasmic accumulation of HuR and its cleavage during myogenesis. Importantly, the cytoplasmic accumulation of HuR was shown to be a crucial event for the myogenic process and is associated to the stabilization function of HuR (66,84,85,93).

Post-translational modification of proteins is known to play an important role in mediating protein-protein interactions. In some instances, it has also been shown to regulate the interactions of RBPs with other proteins during myogenesis (117–123). Indeed, HuR is known to collaborate or compete with other RBPs to regulate the stability of target messages (85–87). As such, since one of the impacts of PARylation is to modulate protein–protein interactions, it is possible that TNKS1-mediated PARylation of HuR during myogenesis modulates its interactions with protein partners to differentially regulate the expression/stability of its mRNA targets. In fact, our group has previously shown that during the early steps of the myogenic process, HuR forms a complex with KSRP to promote the degradation of the *NPM* mRNA and that this event is required for the commitment of muscle cells to the differentiation process (39). The fact TNKS1-mediated PARylation is also important for this event, raises the possibility that this modification is also important for the association of HuR with protein ligands such as KSRP.

Our study, therefore, uncovers the importance of TNKS1-mediated PARylation as a key determinant of skeletal muscle formation. This outcome occurs, in part, due to the PARylation of RBPs, such as HuR, that regulate the expression of mRNAs encoding factors that control the fate of skeletal muscle.

## Supplementary Material

gkae059_Supplemental_Files

## Data Availability

Mass spectrometry data are publicly available as of the date of publication (https://www.proteomexchange.org/, PXD039388). Any additional information required to reanalyze the data reported in this paper is available from the corresponding contact upon request.
