# Supplementary material for: Tankyrase-1 regulates RBP-mediated mRNA turnover to promote muscle fiber formation
Source: Nucleic Acids Res. 2024 Feb 7;52(7):4002–20. doi: 10.1093/nar/gkae059 (PMC11040007; doi:10.1093/nar/gkae059)
Supplement: gkae059_Supplemental_Files [file gkae059_supplemental_files.zip › Supplementary Figure Legends.docx]

**Supplementary Figure Legends**

**Supplementary Figure S1. A)** Immunofluorescence images showing C2C12 cells at different time points of the differentiation process (EXP to Day 3). Immunofluorescence staining was performed with an antibody against Myoglobin & MyHC. DAPI was used to stain nuclei. (Scale bars, 20µm). (**Bottom panel**) Quantification of the fusion index for the C2C12 cells described in A. The fusion index was calculated as the ratio of the nuclei number in myotubes versus the total number of nuclei. **B)** Phase contrast images of C2C12 cells at different time points of the differentiation process. Images of a single field are shown and represent three independent experiments. (Scale bars, 200µm). Data shown are presented +/- the s.e.m. of 3 independent experiments.

**Supplementary Figure S2.** C2C12 muscle cells were transfected with scramble control (siCTRL) or with two different sets of siRNAs specific for the different PARPs (*PARP1*, *PARP2*, *PARP3*, *PARP4*, *TNKS1*, and *TNKS2*). The second set of siRNAs are labeled with the suffix “-B” for clarification purposes. Total RNA and protein lysates were prepared from these cells on Day 0 and mRNA and protein levels of the corresponding PARPs were determined by RT-qPCR and/or Western blot to determine silencing efficiency. **A,C**) mRNA levels were standardized against *RPL32* mRNA and expressed relative to siCTRL conditions. **B,D)** (**Left Panels**) Protein levels were assessed relative to β-actin (Loading control). (**Right panels**) Quantification of the western blots from left panels. Values were quantified using ImageJ and normalized to β-actin. Data shown are presented +/- the s.e.m. of 3 independent experiments with *P <0.05, **P <0.01, ***P <0.001 by unpaired *t*-test.

**Supplementary Figure S3. (A)** Phase contrast images of C2C12 muscle cells transfected with scramble control (siCTRL) or with a second siRNAs specific for the different PARPs (*PARP1*, *PARP2*, *PARP3*, *PARP4*, *TNKS1*, and *TNKS2*). (Scale bars, 200µm). **B)** (**Top Panel**) Immunofluorescence images were taken of cells treated with the siRNAs described above 3 days after induction of differentiation. Immunofluorescence staining was performed with an antibody against Myoglobin & MyHC. DAPI was used to stain nuclei. Images of a single field are shown and represent three independent experiments. (Scale bars, 20µm). **(Bottom Panel)** Quantification of the fusion index for the C2C12 cells described in top panel. The fusion index was calculated as the ratio of the nuclei number in myotubes versus the total number of nuclei. **C)** Primary myoblasts were transfected with siCTRL or siTNKS1. Total RNA was isolated from myotubes on Day 3 post-induction of differentiation and *TNKS1* mRNA levels were determined by RT-qPCR. Levels were standardized against *GAPDH* mRNA and expressed relative to siCTRL conditions to determine silencing efficiency. Data shown in figure S3 are presented +/- the s.e.m. of 3 independent experiments with *P <0.05, by unpaired *t*-test.

**Supplementary Figure S4. A)** Total cell extracts were prepared from differentiating C2C12 cells (Day 2) that were treated with 10uM XAV939 or DMSO as a control. These extracts were used for western blot analysis with antibodies against pADPr or α-tubulin (loading control) (**Left panel**). **(Right panel)** Histogram representation of the quantification of the western blot (right panel). Values were quantified using ImageJ normalized to tubulin and shown relative to DMSO treated control condition. **B)** Immunofluorescence experiments on differentiating C2C12 myoblasts treated with 10uM XAV939 or DMSO. Staining was performed with antibodies against known markers of muscle fiber formation, MyHC, and myoglobin. DAPI was used to stain nuclei. Images of a single field are shown and represent three independent experiments. (Scale bars, 20µm). (**C**) Quantification of the fusion index for the C2C12 cells shown in left panel. The fusion index was calculated as the ratio of the nuclei number versus the total number of nuclei. Data shown in figure S4 are presented +/- the s.e.m. of 3 independent experiments with **P <0.01, by unpaired *t*-test.

**Supplementary Figure S5.** C2C12 muscle cells were transfected with scramble control (siCTRL) or with two different siRNAs specific for TNKS1 (siTNKS1 and siTNKS1-B). Total RNA and protein lysates were prepared from these cells on Day 0. **A)** mRNA levels of TNKS2 were assessed by RT-qPCR. mRNA levels were standardized against *RPL32* mRNA and plotted relative to siCTRL condition. **B)** **(Left panel)** Western blot experiments was performed using antibodies specific against PARP1, TNKS1 and α-tubulin (Loading control). (**Right panel**) Quantification of the western blots in left panel. Values were quantified using ImageJ and normalized to tubulin. Data shown are presented +/- the s.e.m. of 3 independent experiments with *P <0.05 by unpaired *t*-test. (**C)** Protein lysates were prepared from C2C12 cells harvested at the EXP phase as well as at Day 0 and D2 of the differentiation process. These lysates were used in Western blot experiments with antibodies specific against PARP1, TNKS1 and α-tubulin (Loading control). (**D**) Histogram representation of the quantification of the western blot shown in C. Values were quantified using ImageJ, normalized to tubulin, and shown relative to the EXP siCTRL treated condition. Data are presented +/- the s.e.m. of 3 independent experiments with *P <0.05, **P <0.01 by unpaired *t*-test.

**Supplementary Figure S6. A) (Left Panel)** Total cell extracts were prepared from C2C12 cells transfected with scrambled control (siCTRL) or siRNA against TNKS1 (siTNKS1) and collected from exponentially growing (EXP) and differentiating C2C12 myoblasts (Day 0 and Day 2). Extracts were used for western blot analysis with antibodies against HuR or α-tubulin (loading control). (**Right panel**) Quantification of the western blot in left panel. Values were quantified using ImageJ and normalized to tubulin. Quantifications are shown relative to the EXP siCTRL treated condition. Data shown are presented +/- the s.e.m. of 3 independent experiments. **B) (Left Panel)** Immunoprecipitation experiments using pADPr or IgG antibodies were performed with extracts from differentiating C2C12 cells (Day 2) treated with 10uM XAV939 or DMSO as a control. The association of HuR to pADPr was determined by western blot analysis. The blot shown is a representation of three independent experiments. (**Right panel**) The levels of immunoprecipitated HuR were normalized to the corresponding IgG sample and plotted relative to the DMSO condition. Data shown are presented +/- the s.e.m. of 3 independent experiments with *P <0.05, **P <0.01, ***P <0.001 by unpaired *t*-test.

**Supplementary Figure S7.** C2C12 muscle cells were transfected with scramble control (siCTRL) or siRNAs specific for TNKS1 (siTNKS-B). Total RNA and protein lysates were prepared from these cells 2 days post-induction of differentiation. **A)** *NPM* (**Left panel**) and *Myogenin* (**Right panel**)*,* mRNA levels were determined by RT-qPCR, standardized against *GAPDH* mRNA, and expressed relative to siCTRL conditions. **B,C)** Total extracts were used for western blot analysis to determine NPM (**B**) and Myogenin (**D**) protein levels. (**Bottom of B,C**) Histogram representation of the quantification of the western blot . Values were quantified using ImageJ, normalized to tubulin, and shown relative to the EXP siCTRL treated condition. **D,E)** RNA-Immunoprecipitation coupled to RT-qPCR experiments was performed using anti-HuR (3A2) and anti-IgG antibodies on total extracts from differentiating C2C12 cells treated with scrambled control (siCTRL) or siRNA against TNKS1 (siTNKS1). (**D**) Western blot assessing the immunoprecipitation of HuR. (**E**) *NPM* and *Myogenin* mRNA levels in the immunoprecipitates were normalized to the corresponding IgG sample and mRNA input. The levels of *NPM* and *myogenin* mRNA in siTNKS1 conditions were plotted relative to siCTRL conditions. **F,G)** Actinomycin D (Act. D) pulse-chase assays were performed using C2C12 myoblasts transfected as described above and treated for various periods of time with Actinomycin D to assess the stability of *NPM* (**F**) and *Myogenin* (**G**) mRNAs. Data shown in Figure 3 are presented +/- the s.e.m. of 3 independent experiments with *P <0.05, **P<0.01, ***P <0.001 by unpaired *t*-test.

**Supplementary Figure S8 A-B)** RNA-Immunoprecipitation coupled to RT-qPCR experiments was performed using anti-HuR (3A2) or anti-IgG antibodies on total extract from differentiating C2C12 cells that were treated with 10uM XAV939 or DMSO as a control. **A)** Western blot assessing the immunoprecipitation of HuR. **B)** The levels of *NPM* and *Myogenin* mRNAs in the immunoprecipitates were normalized to the corresponding IgG sample and plotted relative to DMSO conditions. **C)** Total RNA was isolated from differentiating C2C12 cells (Day 2) treated with 10uM XAV939 or DMSO as a control. The levels of *NPM* (top panel) and *Myogenin (bottom panel)* mRNAs were determined by RT-qPCR, standardized against *GAPDH* mRNA, and expressed relative to DMSO conditions. **D-E)** Lysates were prepared from C2C12 cells as described above and used for western blot analysis (Upper panels) using antibodies against NPM (**D**), Myogenin (**E**), or α-tubulin (Loading control). (**Bottom panels**) Quantification of the western blots in top panels. Values were quantified using ImageJ and normalized to tubulin, then to the DMSO condition. Data shown are presented +/- the s.e.m. of 3 independent experiments with *P <0.05, **P <0.01, ***P <0.001 by unpaired *t*-test.

**Supplementary Figure S9. A)** Immunofluorescence images showing the localization of HuR in differentiating (Day 2) muscle myoblasts treated with siCTRL or siTNKS1-B. Immunofluorescence staining was performed with an antibody against HuR (3A2). DAPI was used to stain nuclei. Images of a single field are shown and represent three independent experiments. (Scale bars, 20µm) **B,C) (B)** Western blot analysis of subcellular localization of C2C12 cells (Day 2) treated with siCTRL or two different siRNAs against TNKS1 (siTNKS1 and siTNKS1-B), using antibodies against HuR, Tubulin (Cytoplasmic control), Histone 3 (Nuclear control) and β-actin (loading control). The blot is representative of three independent experiments. (**C**) Quantification of the western blots. Values were quantified using ImageJ and normalized to β-actin. Data shown are presented +/- the s.e.m. of 3 independent experiments with p-values calculated by unpaired *t*-test with *P <0.05.

**Supplementary Figure S10.** Immunofluorescence images showing the localization of HuR in differentiating (Day 2) muscle myoblasts treated with 10uM XAV939 or DMSO (used as a control). Immunofluorescence staining was performed with an antibody against HuR. DAPI was used to stain nuclei. Images of a single representative field are shown and are representations of three independent experiments. (Scale bars, 20µm)

**Supplementary Figure S11.** Coomassie staining of GST, GST-HuR^WT^ and GST-HuR^G224D^ used in Ribosylation assay in Figure 6D.

**Supplementary Figure S12.** **A)** Knockdown of HuR and overexpression of GFP, GFP-HuR^WT^ and GFP-HuR^G224D^ were assessed by western blot analysis using antibodies against HuR, GFP, or α-tubulin (loading control). The blot is representative of three independent experiments. **(B)** Phase contrast images of cells described in A assessing rescue of the myogenic phenotype in HuR knockdown cells. (Scale bars, 50µm)

**Supplementary Table 1.** pADPr-immunoprecipitation - Mass Spectrometry Table (Unique protein list-total list)

**Supplementary Table 2.** pADPr-immunoprecipitation - Mass Spectrometry Table (Sheet 1: Proteins classified by Panther-GO-Molecular function) (Sheet 2: Pie Chart)

**Supplementary Table 3.** List of catalog numbers of primers for siRNAs acquired from companies and their exact sequences for the customized ones against HuR. List of the PCR primers used throughout the paper as well as the antibodies and their catalog numbers.

**Supplementary Table 3**

| **siRNAs** | | |
| --- | --- | --- |
| **Targeted Gene** | **siRNA** | **siRNA-B** |
| PARP1 | AM4390825 (s1097) | 10620318 Parp1IMSS201785 |
|  | AM4390815 (s62054) |  |
| PARP2 | AM16704 (111561) | AM16704 (162376) |
| PARP3 | AM51331 (111564) | AM4390771 (s108205) |
| PARP4 | AM16704 (7278) | AM16704(s78641) |
| Tnks1 | AM16706 (13634) | AM4390815(s75316) |
| Tnks2 | AM16708A (32532) | AM4390815(s78641) |
| HuR | AM4390815 (s67964) |  |
|  | 3'UTR custom: 5'CAG AAA CAU UUG AGC AUU GUA-dTdT-3' |  |
| **qPCR primers** | | |
| **Targeted Gene** | **Forward** | **Reverse** |
| PARP1 | F: 5'-CCC AGG GTC TTC GAA TAG-3' | R: 5'-AGC GTG CTT CAG TTC ATA C-3' |
|  | F:5'-AGT CAA TCT CCA GAG GGT AGA A-3' | R: 5'- AAG GAG AGC AGG TAC TGG AT -3' |
| PARP2 | F: 5'-GGA AGG CGA GTG CTA AAT GAA-3' | R: 5'-AAG GTC TTC ACA GAG TCTCGA TTG-3' |
| PARP3 | F: 5'-AGG GTG CAT CAC ACT CAA GG-3' | R: 5'-AGA TTT GCT GGT CTC TGG GC-3' |
| PARP4 | F: 5'-TCT ATC CTG CTT CCT CGC CT-3' | R: 5'-TGG CAG TGT TTC CTA CTC CC-3' |
|  | F: 5' TAA GTG CAT CAC GGA CAG TAA A-3' | R: 5'- CACTCT GGT GTG CTG GAT ATT-3' |
| Tnks1 | F: 5'-GCA ACA GAT TCT GAG CGA GAG-3' | R: 5'-TCA ACG ACA GAC ACA CGG TT-3' |
| Tnks2 | F: 5'-CCA GGA GTG GCA ATG AGG AA-3' | R: 5'-TGT AGT GGT ACC AGA TCC CCT-3' |
|  | F: 5'- GGG TGT CCA GTT CAC AAA GA-3' | R: 5'-AAG CCT GTT CTC CTC TGT AAA TAA-3' |
| HuR | F: 5'-ATG GTC ATA AAC CCC CAG GT-3' | R: 5'-GGG GAC ATT GAC ACC AGA AA-3' |
| Myogenin | F: 5'-CTA CAG GCC TTG CTC AGC TC-3' | R: 5'-AGA TTG TGG GCG TCT GTA GG-3' |
| MyoD | F: 5'-CGA CAC CGC CTA CTA CAG TG-3' | R: 5'-TTC TGT GTC GCT TAG GGA TG-3' |
| NPM | F: 5'-CCG AGA TCA AAG GGT CAA GA-3' | R: 5'-TCT TGA ATA GCC TCC TGG TCA-3' |
| GAPDH | F: 5'-AAG GTC ATC CCA GAG CTG AA-3' | R: 5'-AGG AGA CAA CCT GGT CCT CA-3' |
| RPL32 | F: 5’- TTCTTCCTCGGCGCTGCCTACGA -3’ | R: 5’-AAC CTT CTC CGC ACC CTG TTG TCA |
| **Antibodies** | | |
| **Protein detected** | | **Catalog number** |
| Poly-ADP-ribose | | Western Blot: 96-10 (Guy Poirier) |
|  |  | IP: Tulips-1020 |
| Tubulin | | Abcam, ab11304 |
| MyHC (MF-20) | | Developmental studies Hybridoma Bank, |
| Myoglobin | | Abcam: ab77232 |
| KSRP | | Bethyl: A302-021A |
| HuR (3A2) | | In house 3A2 (Imed Gallouzi) |
| NPM (B23) | | Sigma: B0556 |
| GFP | | Clonetech (JL-8): 632381 |
| Mouse and Rabbit Secondary IgG | | Jackson ImmunoResearch |
| Goat anti-rabbit (594) | | ThermoFisher: A11072 |
| Goat anti-mouse (488) | | ThermoFisher: A11029 |
| TRN2 | | BioCan |
| Myogenin (F5D) | | Invitrogen MA5-11486 |
| Tankyrase1 (TNKSI) | | Proteintech, 18030-1-AP |
| PARP1 (46D11) | | Cell Signaling, mAb #9532 |
| PARP2 | | Invitrogen, PA5-101335 |
| PARP3 | | Proteintech, 11289-1-AP |
| PARP4 | | Invitrogen, PA5-76368 |
| Tankyrase 2 (TNKS2) | | Abcam, ab155545 |
| Histone H3 | | Abcam, ab8898 |
| Beta-actin (BA3R) | | Invitrogen, MA5-15739 |
