# Supplementary material for: Tankyrase-1 regulates RBP-mediated mRNA turnover to promote muscle fiber formation
Source: Nucleic Acids Res. 2024 Feb 7;52(7):4002–20. doi: 10.1093/nar/gkae059 (PMC11040007; doi:10.1093/nar/gkae059)
Supplement: gkae059_Supplemental_Files [file gkae059_supplemental_files.zip › Supplementay_Fig_Mubaid_et_al_FINAL_Dec_12_2023.pdf]

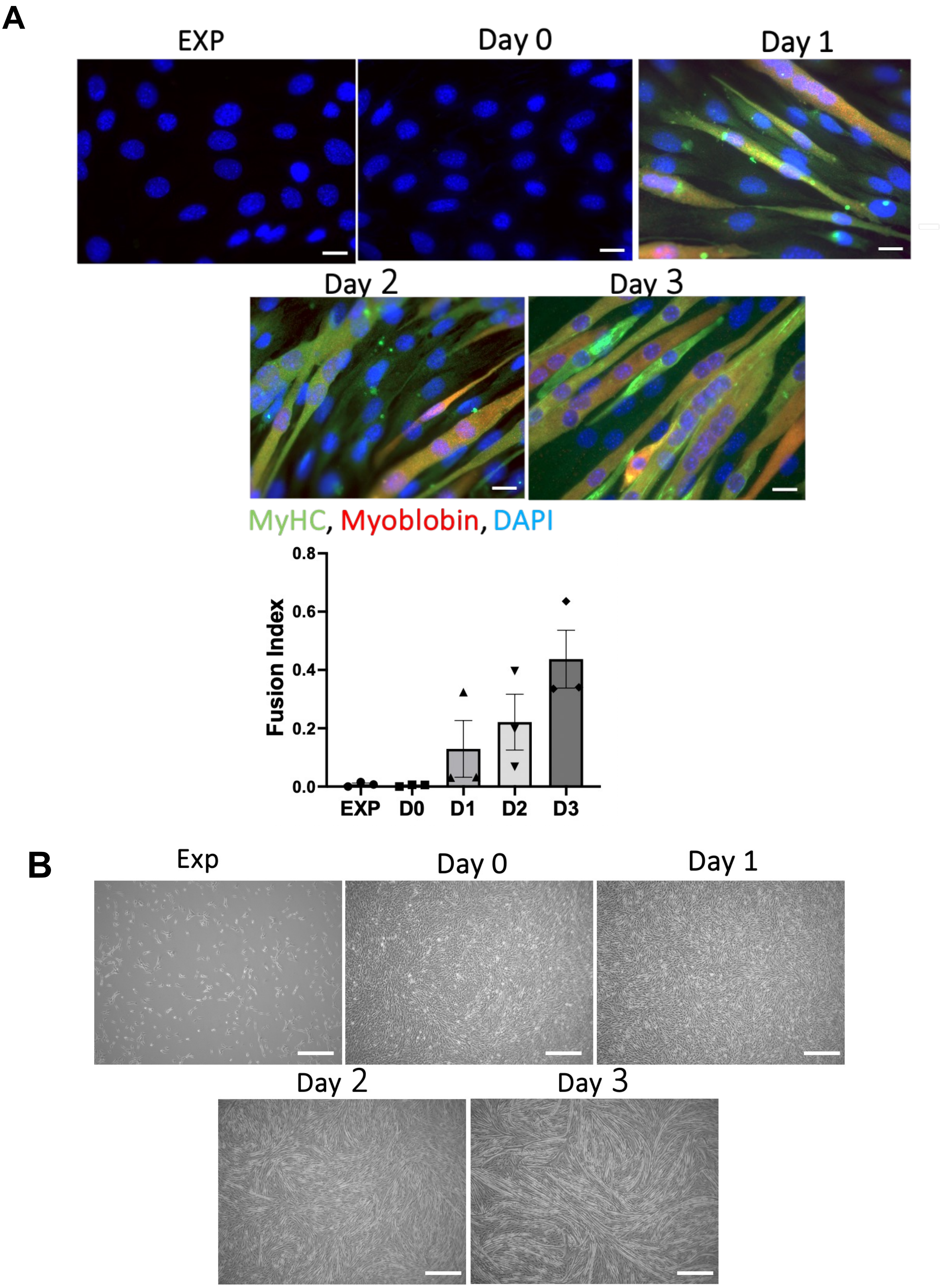

Supplementary Figure S1

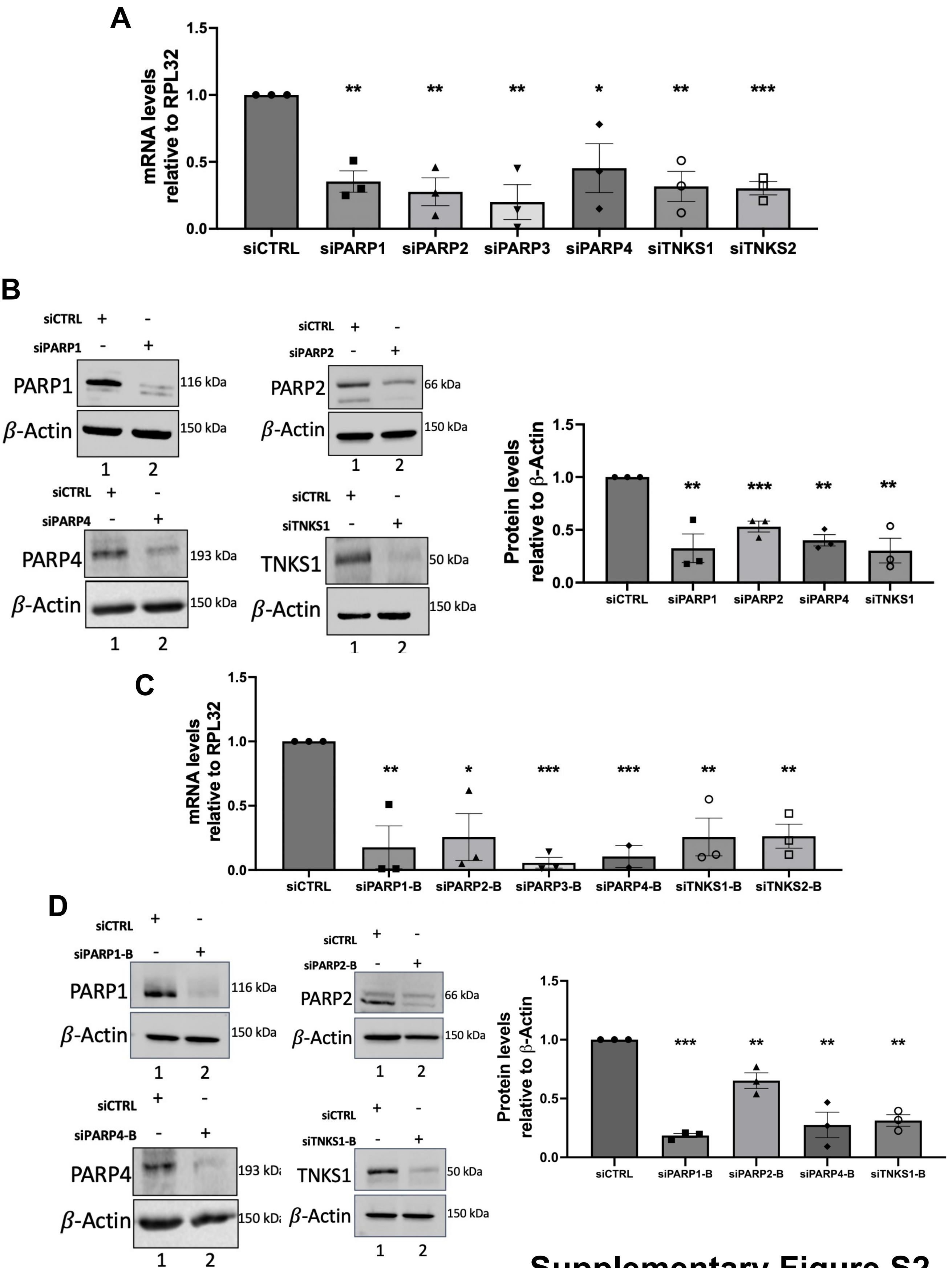

Supplementary Figure S2

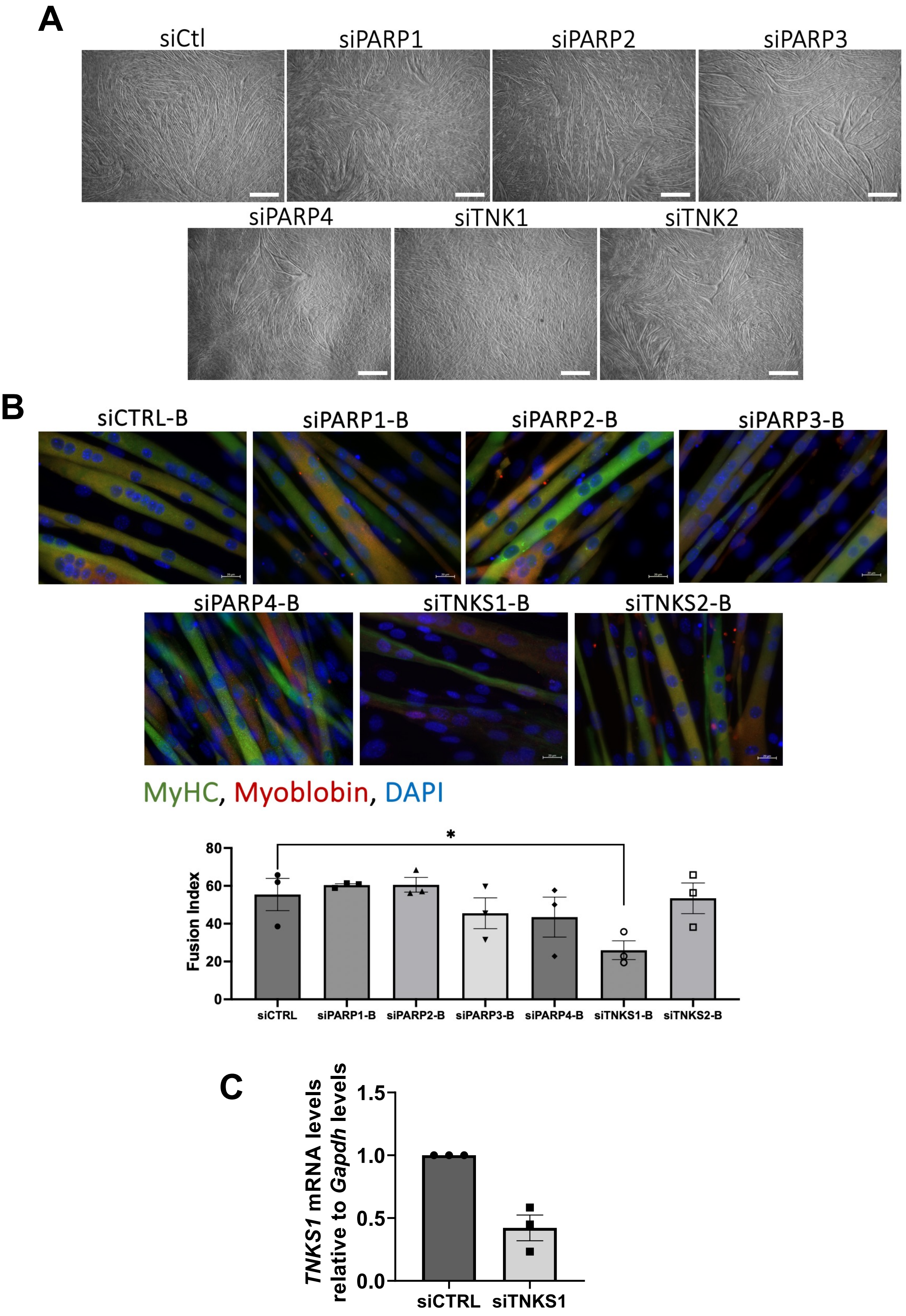

Supplementary Figure S3

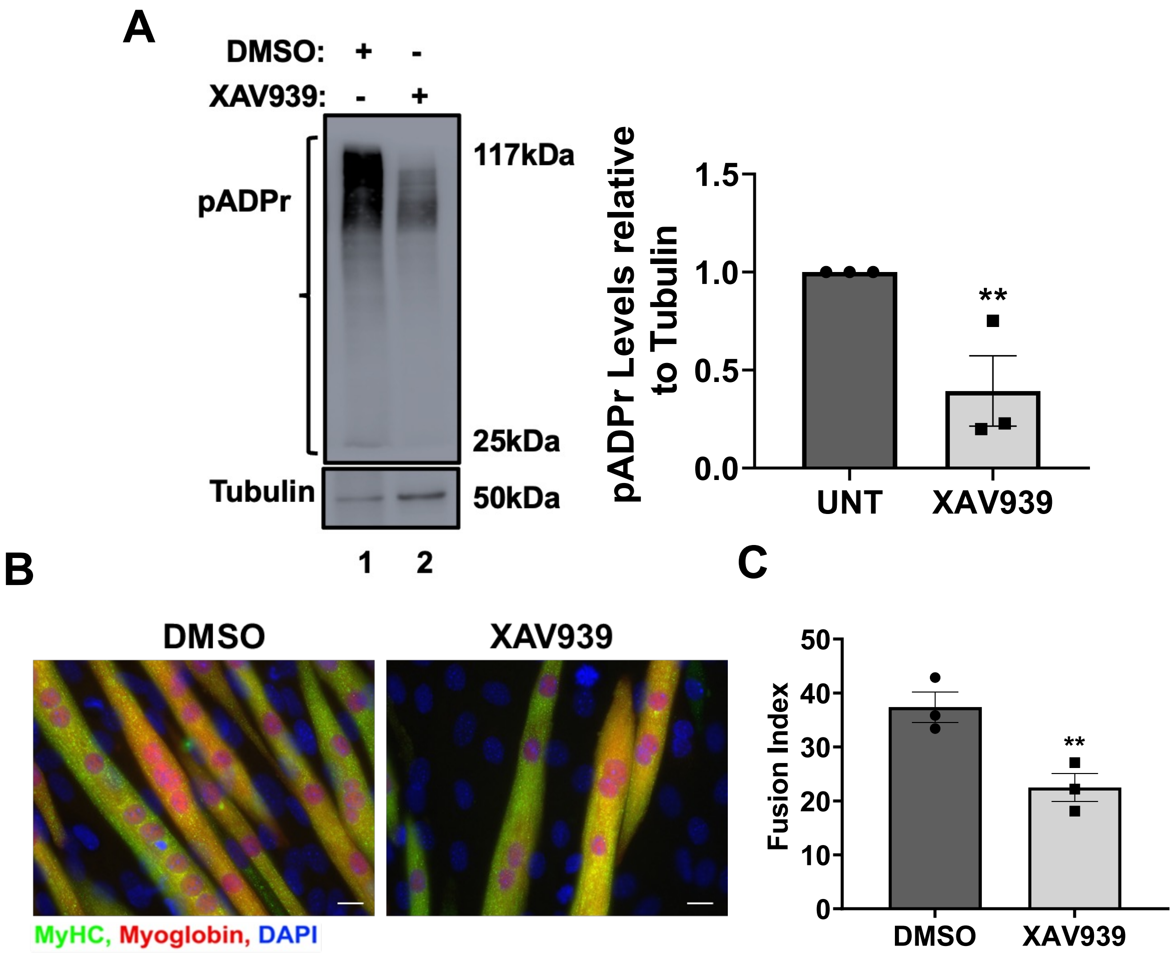

Supplementary Figure S4

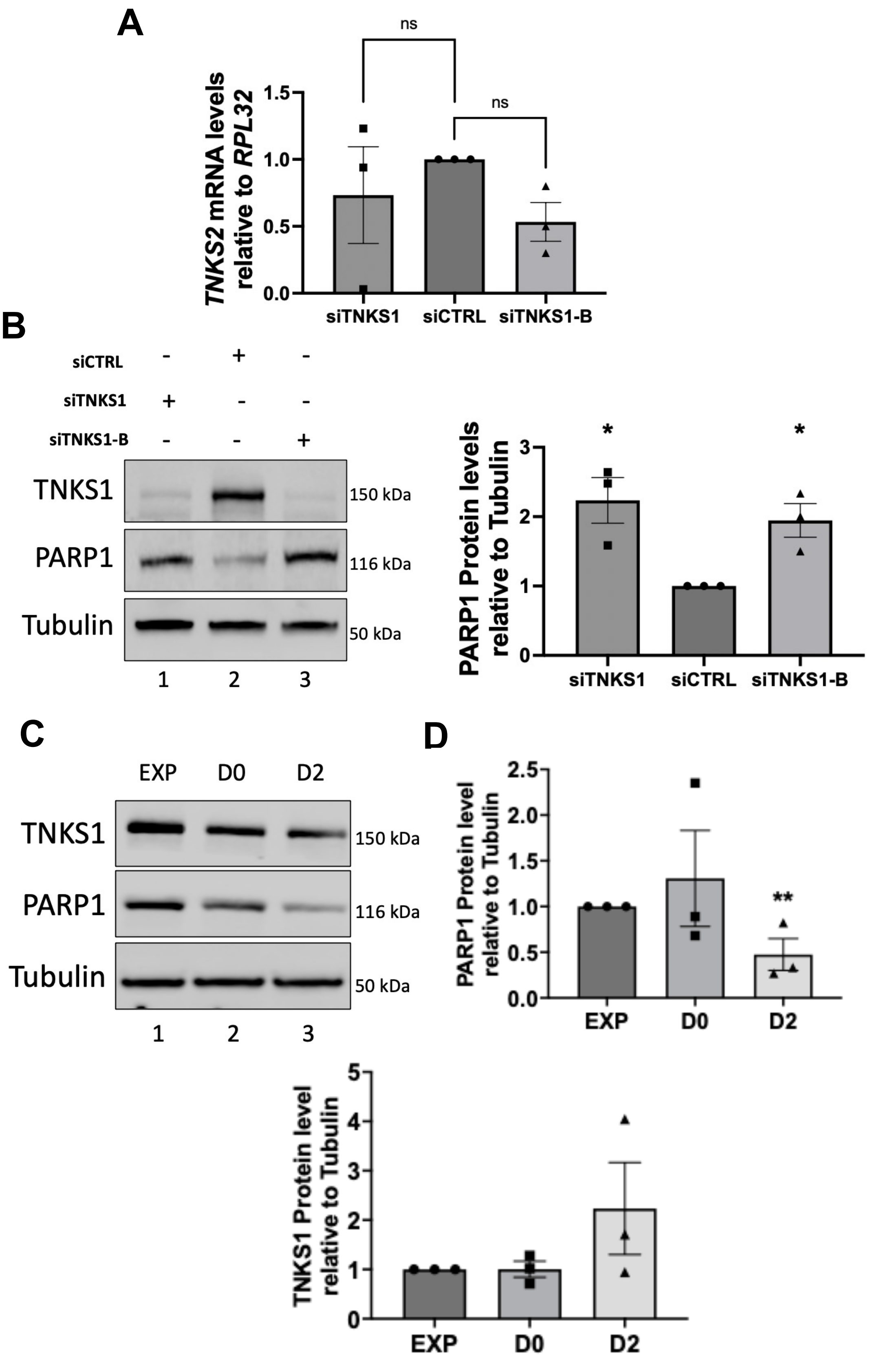

Supplementary Figure S5

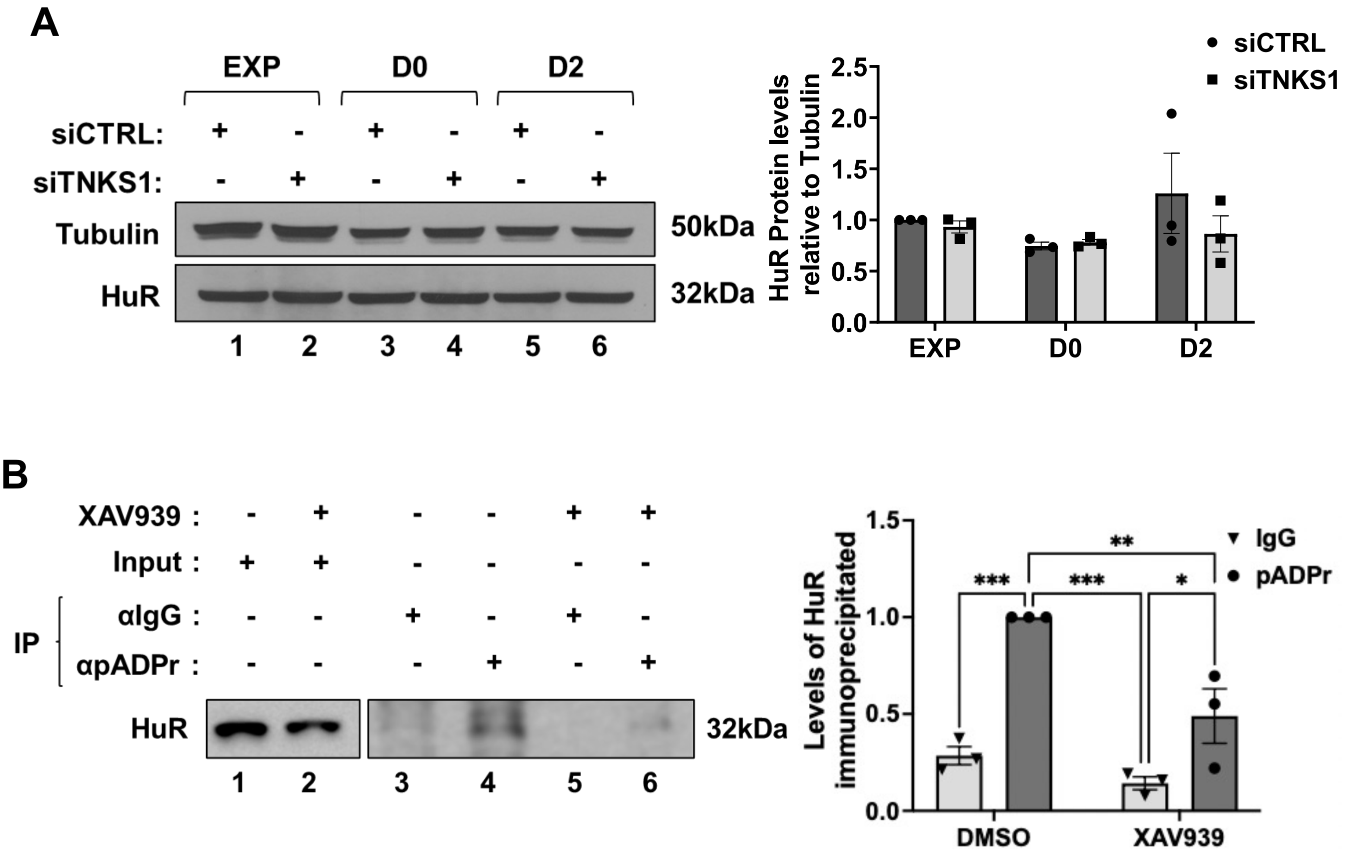

Supplementary Figure S6

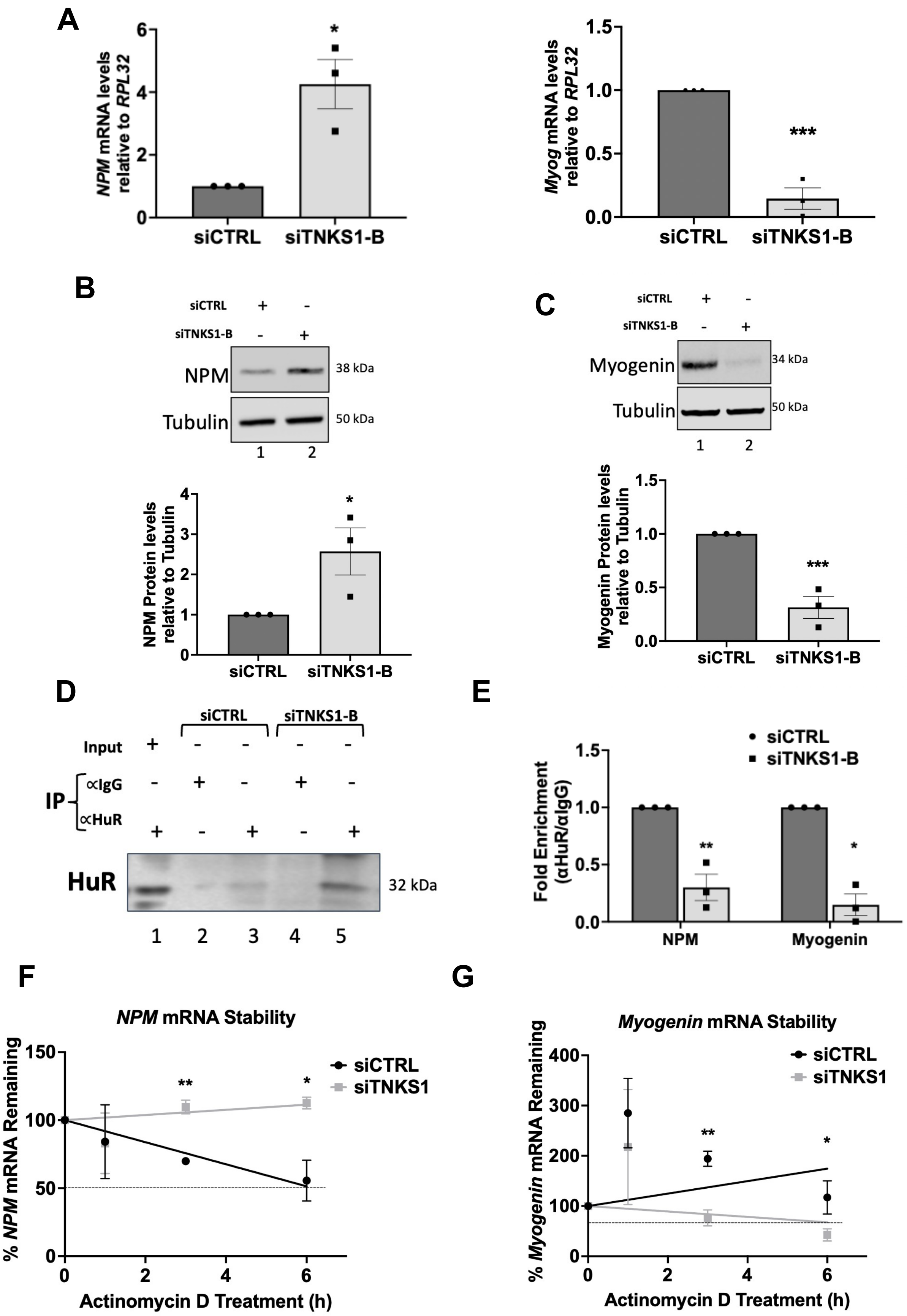

Supplementary Figure S7

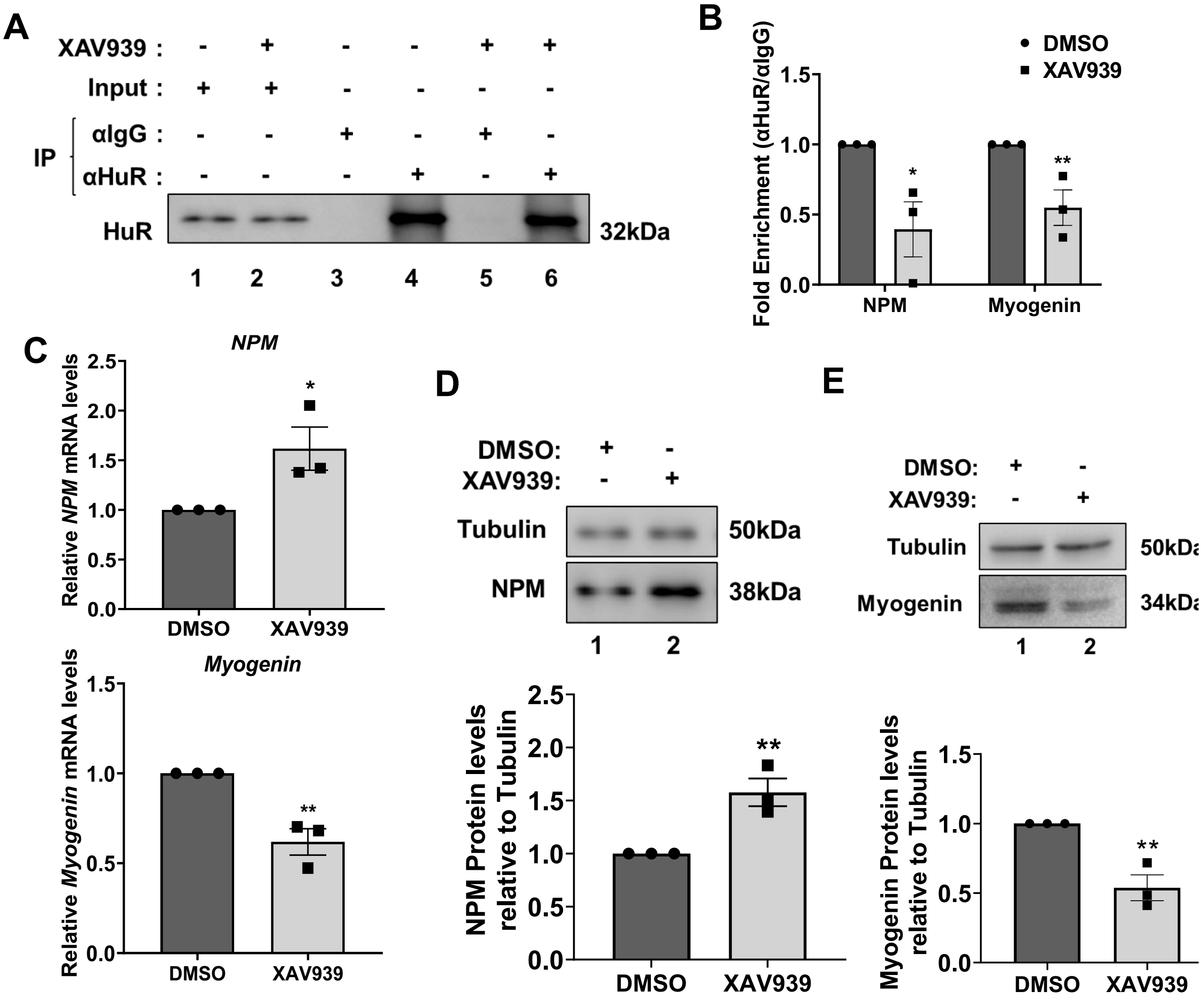

Supplementary Figure S8

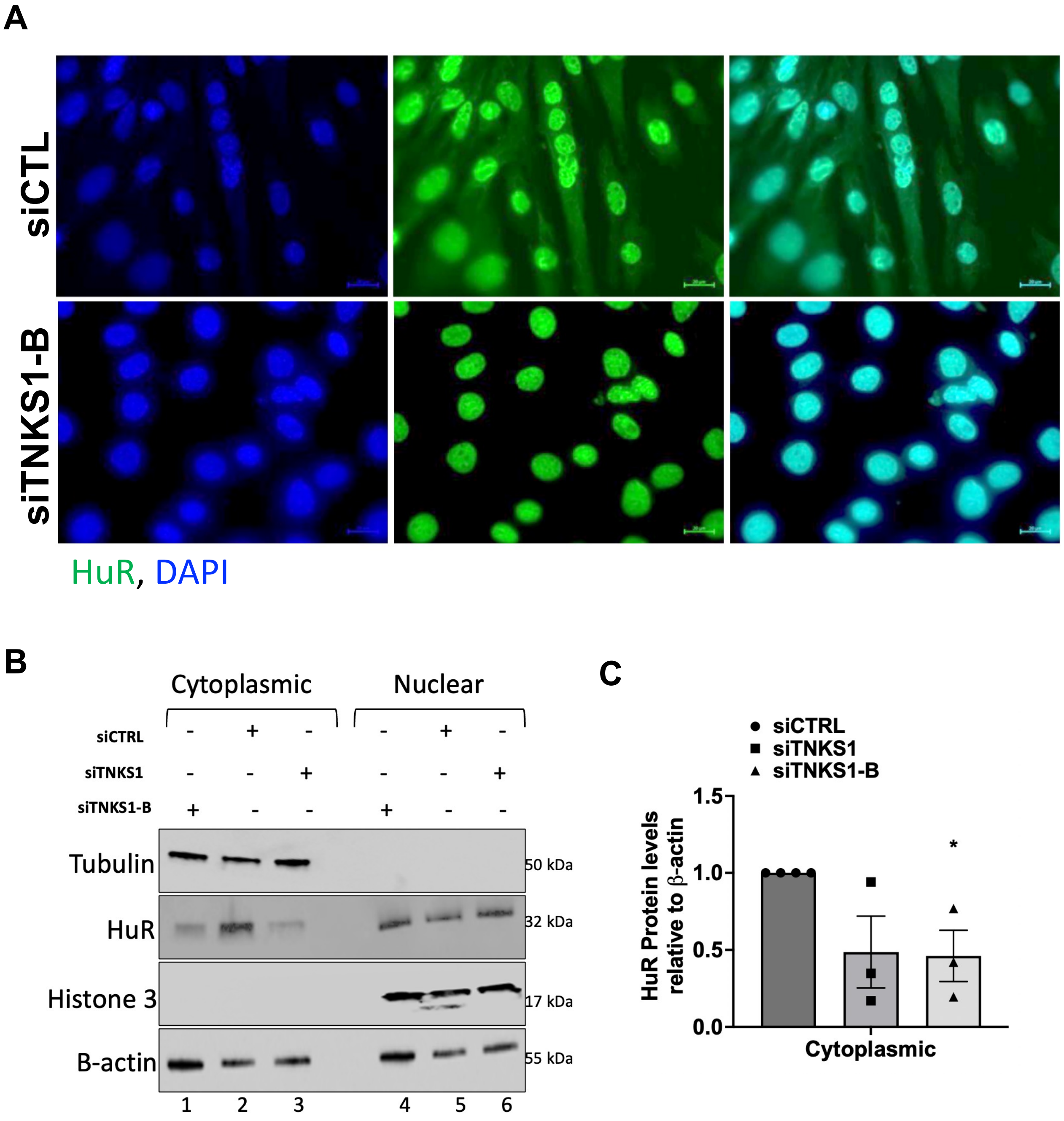

Supplementary Figure S9

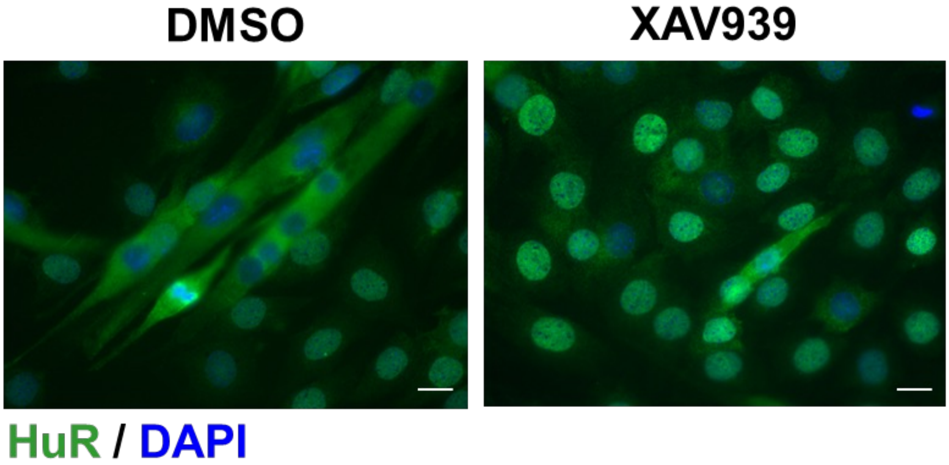

Supplementary Figure 10

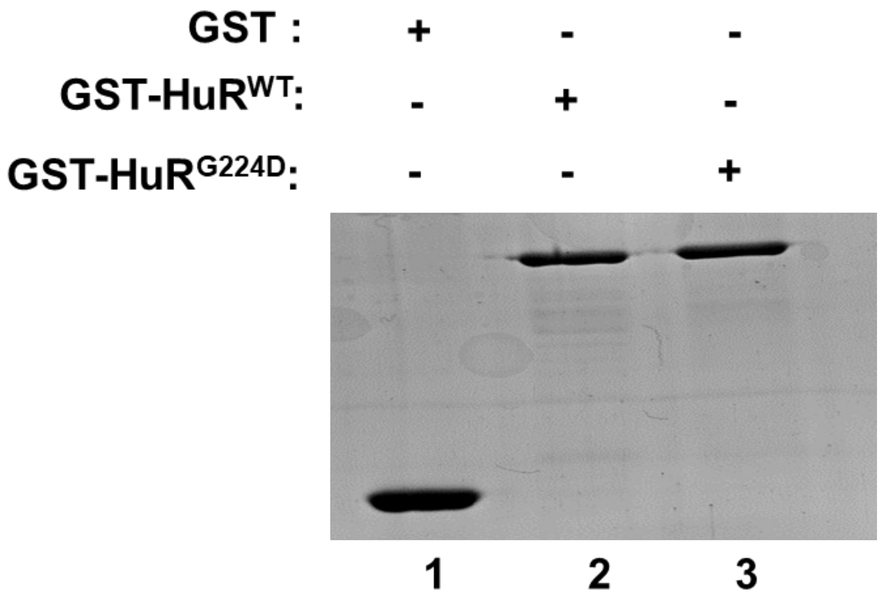

Supplementary Figure S11

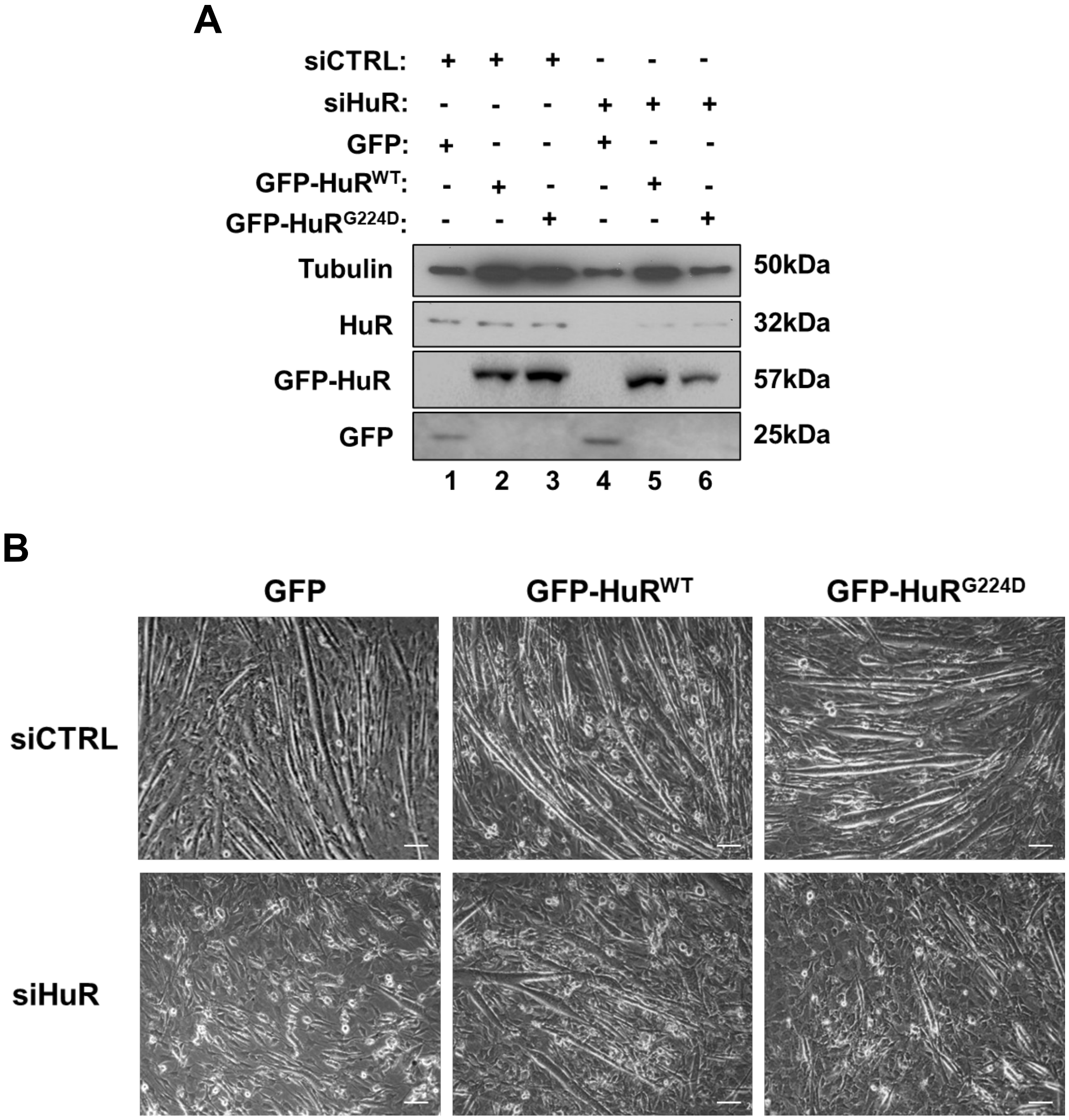

Supplementary Figure S12
